# PHD3 regulates glucose metabolism by suppressing stress-induced signalling and optimising gluconeogenesis and insulin signalling in hepatocytes

**DOI:** 10.1038/s41598-018-32575-z

**Published:** 2018-09-24

**Authors:** Hiroyuki Yano, Mashito Sakai, Toshiya Matsukawa, Takashi Yagi, Takao Naganuma, Masaru Mitsushima, Satoshi Iida, Yuka Inaba, Hiroshi Inoue, Hiroyuki Unoki-Kubota, Yasushi Kaburagi, Shun-ichiro Asahara, Yoshiaki Kido, Shiro Minami, Masato Kasuga, Michihiro Matsumoto

**Affiliations:** 1 Department of Molecular Metabolic Regulation, Diabetes Research Center, Research Institute, National Center for Global Health and Medicine, Tokyo, 162-8655 Japan; 2 Department of Bioregulation, Institute for Advanced Medical Sciences, Nippon Medical School, Kawasaki, 211-8533 Japan; 3 Metabolism and Nutrition Research Unit, Innovative Integrated Bio-research Core, Institute for Frontier Science Initiative, Kanazawa University, Kanazawa, 920-8641 Japan; 4 Department of Diabetic Complications, Diabetes Research Center, Research Institute, National Center for Global Health and Medicine, Tokyo, 162-8655 Japan; 5 Division of Diabetes and Endocrinology, Department of Internal Medicine, Kobe University Graduate School of Medicine, Kobe, 650-0017 Japan; 6 Division of Metabolism and Disease, Department of Biophysics, Kobe University Graduate School of Health Sciences, Kobe, 654-0142 Japan; 7 National Center for Global Health and Medicine, Tokyo, 162-8655 Japan

## Abstract

Glucagon-mediated gene transcription in the liver is critical for maintaining glucose homeostasis. Promoting the induction of gluconeogenic genes and blocking that of *insulin receptor substrate* (*Irs*)2 in hepatocytes contributes to the pathogenesis of type 2 diabetes. However, the molecular mechanism by which glucagon signalling regulates hepatocyte metabolism is not fully understood. We previously showed that a fasting-inducible signalling module consisting of general control non-repressed protein 5, co-regulator cAMP response element-binding protein binding protein/p300-interacting transactivator with Glu/Asp-rich carboxy-terminal domain 2, and protein kinase A is required for glucagon-induced transcription of gluconeogenic genes. The present study aimed to identify the downstream effectors of this module in hepatocytes by examining glucagon-induced potential target genes. One of these genes was *prolyl hydroxylase domain* (*PHD*)3, which suppressed stress signalling through inhibition of the IκB kinase–nuclear factor-κB pathway in a proline hydroxylase-independent manner to maintain insulin signalling. PHD3 was also required for peroxisome proliferator–activated receptor γ coactivator 1α-induced gluconeogenesis, which was dependent on proline hydroxylase activity, suggesting that PHD3 regulates metabolism in response to glucagon as well as insulin. These findings demonstrate that glucagon-inducible PHD3 regulates glucose metabolism by suppressing stress signalling and optimising gluconeogenesis and insulin signalling in hepatocytes.

## Introduction

Blood glucose homeostasis is primarily maintained by the coordinated action of two pancreatic hormones, glucagon and insulin. During fasting, glucagon is secreted from islet α cells in response to hypoglycaemia, and stimulates hepatic glucose production through gluconeogenesis and glycogenolysis^1^. The glucagon receptor (GcgR)–cyclic (c)AMP–protein kinase (PK)A pathway promotes hepatic gluconeogenesis by inducing expression of the gluconeogenic genes *G6pc* and *Pck1* (encoding the catalytic subunit of glucose-6-phosphatase and phosphoenolpyruvate carboxykinase, respectively)^2^. Such induction is thought to be mediated through the coordination of hormone-dependent assembly of transcriptional machinery^3–6^ and epigenetic changes^7,8^ at gene promoters. The former includes transcription factor–transcriptional coactivator complexes; for instance, assembly of the cAMP response element-binding protein (CREB)–CREB-regulated transcriptional coactivator (CRTC)2 complex is triggered by PKA-dependent CREB phosphorylation and CRTC2 dephosphorylation, which promotes the recruitment of the histone acetyltransferase CREB-binding protein (CBP, also known as Kat3a), thereby activating peroxisome proliferator-activated receptor (PPAR)-γ coactivator (PGC)-1α transcription^5^. PGC-1α is activated by Sirtuin (Sirt)1-mediated deacetylation^9^ and inhibited by general control non-repressed protein (GCN)5 (also known as Kat2a)-mediated acetylation^10^, and acts in association with the transcription factors forkhead box (Fox)O1^11^ and hepatocyte nuclear factor (HNF)-4α^12,13^ to synergistically promote gluconeogenesis.

In a postprandial state, nutrient intake stimulates insulin secretion from islet β cells, which promotes glucose uptake and suppresses glucagon secretion and hepatic glucose production. This inhibitory effect is mainly mediated through the insulin receptor (IR)–IR substrate (IRS)-1/2–phosphoinositide 3-kinase signalling pathway^14,15^. Glucagon signalling is also thought to regulate hepatic insulin signalling. *Irs2* gene transcription is induced by the GcgR–PKA–CRTC2–CREB pathway^16^ in concert with FoxO1^17^. Fasting-induced upregulation of IRS-2 enhances postprandial insulin signalling and thereby blocks gluconeogenesis^16–18^. Thus, glucagon-regulated hepatic gene transcription is critical for maintaining euglycaemia in both fasting and postprandial states, but the factors mediating this process have not been fully elucidated.

In obese and diabetic individuals, chronic nutrient excess alters the production of pro- and anti-inflammatory adipokines, cytokines, and lipid mediators that activate a vast array of stress signalling pathways through c-Jun N-terminal kinase (JNK)^19^, inhibitor of nuclear factor (NF)-κB (IκB) kinase (IKK)^20^, and mechanistic target of rapamycin (mTOR). These pathways cooperatively induce chronic low-grade tissue inflammation and impair insulin action via kinase-dependent serine/threonine phosphorylation of IRS^21^. In the liver, enhanced glucagon and impaired insulin signalling promote hepatic gluconeogenesis through induction of gluconeogenic genes. Both glucagon and stress signalling pathways are simultaneously activated under these pathological conditions, although it is unclear how they crosstalk to regulate hepatocyte metabolism.

We previously reported that the transcriptional co-regulator CBP/p300-interacting transactivator with Glu/Asp-rich carboxy-terminal domain (CITED)2 is critical for PGC-1α-induced gluconeogenesis; CITED2 binds to GCN5 and inhibits its acetylation of PGC-1α, leading to activation of the latter^22^. We also showed that CITED2 forms a fasting-induced signalling module with GCN5 and PKA in which GCN5 is phosphorylated by PKA, driving a GCN5 substrate switch from PGC-1α to histone H3^8^. Deacetylated PGC-1α coactivates FoxO1 and HNF-4α; GCN5-dependent acetylation of histone H3 at Lys9 induces further epigenetic changes associated with active gene transcription—such as acetylation of histone H3 at Lys27 and histone H3 Lys4 trimethylation—at gluconeogenic gene promoters^8^. The gluconeogenic program is fully activated by this module-mediated integration of coactivation and epigenetic changes.

To clarify the physiological role of this module in the regulation of hepatocyte metabolism, we screened glucagon-inducible genes whose expression is inhibited by depletion of CITED2, GCN5, and PGC-1α as potential targets of this module. We identified *prolyl-hydroxylase domain* (*Phd*)3 as a potential target gene and demonstrated through loss- and gain-of-function approaches that PHD3 suppresses various stress signalling pathways through inhibition of NF-κB—a master regulator of stress response—in a proline hydroxylase-independent manner, thereby maintaining insulin signalling. On the other hand, PHD3 was also required for PGC-1α-induced gluconeogenesis in a proline hydroxylase-dependent manner, suggesting a critical role in the regulation of metabolism in response to glucagon as well as insulin. These findings indicate that glucagon-inducible PHD3 regulates glucose metabolism by inhibiting stress signalling and promoting gluconeogenesis and insulin signalling in hepatocytes.

## Results

### PHD3 expression is regulated by glucagon–cAMP–PKA signalling in hepatocytes

**Figure 1:**
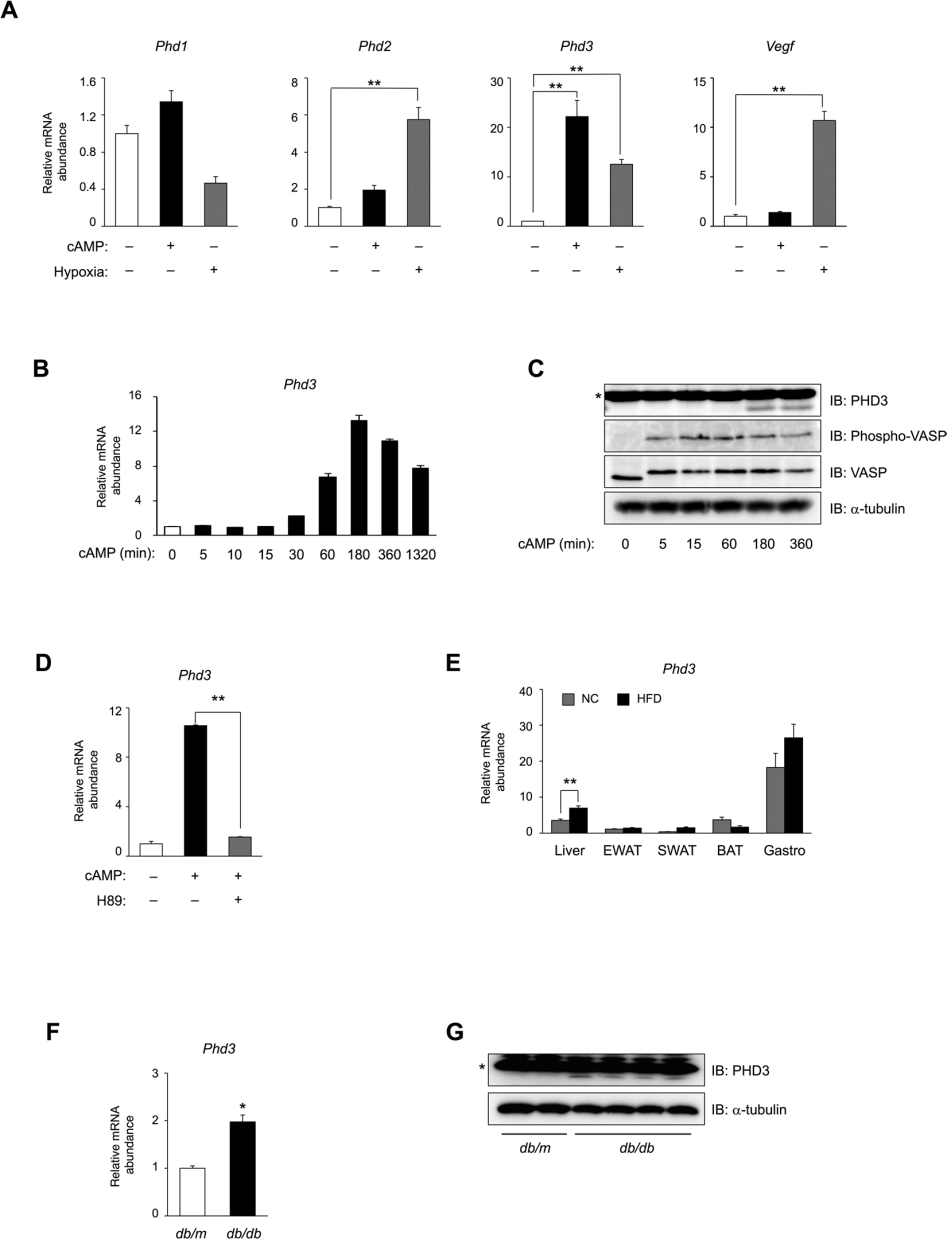
*Phd3* expression is induced by glucagon–cAMP–PKA signalling in mouse hepatocytes. (**A**) qRT-PCR detection of mRNA levels of three PHD isoforms and *Vegf* in primary hepatocytes with or without exposure to pCPT-cAMP (100 μM, 6 h) or hypoxia (1% O_2_, 6 h). (**B**,**C**) qRT-PCR (**B**) and immunoblot (**C**) analyses of PHD3 in primary mouse hepatocytes incubated in the absence or presence of 100 μM pCPT-cAMP for indicated times. Cell lysates were subjected to immunoblot analysis of PHD3, Ser157-phosphorylated VASP, total VASP, or α-tubulin. (**D**) qRT-PCR analysis of *Phd3* mRNA level in primary hepatocytes with or without exposure to pCPT-cAMP (100 μM, 6 h) and with or without pre-treatment with H89 (20 μM, 30 min). (**E**) qRT-PCR analysis of *Phd3* mRNA level in various tissues of C57BL/6 J mice fed normal chow (NC) or a high-fat diet (HFD) for 20 weeks in the fed state. BAT, brown adipose tissue; EWAT, epididymal white adipose tissue; Gastro, gastrocnemius muscle; SWAT, subcutaneous white adipose tissue. (**F**,**G**) qRT-PCR analysis (**F**) and immunoblot analysis (**G**) of PHD3 in the liver of *db*/*db* and *db*/*m* (control) mice fed NC at 8 weeks of age after food deprivation for 16 h. α-Tubulin served as the loading control for immunoblotting. *Non-specific. Complete immunoblots are presented in Supplementary Fig. S7. Quantitative data are shown as mean ± SEM (n = 3 (**A**,**B**,**D**), 7 (**E**), or 6 (**F**)); results in (**A**–**D**) are representative of at least two independent experiments. Differences between groups were evaluated by ANOVA followed by Bonferroni’s post hoc test (**A**,**D**) or with the unpaired Student’s t test (**E**,**F**). *P < 0.05, **P < 0.01 vs. control or as indicated.

The PHD family of oxygen-dependent prolyl hydroxylases comprises three paralogues (PHD1–3) in mammals and regulates the activity of hypoxia-inducible factor (HIF)-1α and -2α. When oxygen is available, PHD hydroxylates the highly conserved proline residues of HIF and promotes von Hippel-Lindau tumour suppressor protein-dependent polyubiquitination and subsequent proteasomal degradation, thereby inhibiting HIF-dependent gene transcription^23,24^. Under hypoxic conditions, HIF is released from this inhibitory hydroxylation and is stabilised, and activates the transcription of genes involved in angiogenesis (e.g. *vascular endothelial growth factor* [*Vegf*]), erythropoiesis, glycolysis, and autophagy^25^. HIF also induces *Phd2* and *Phd3* mRNA expression in a negative feedback loop in HeLa cells^26^. In primary cultured mouse hepatocytes, hypoxia induced mRNA expression of *Phd2*, *Phd3*, and *Vegf* (Fig. 1A). Exposure to glucagon (Supplementary Fig. S1A) or a cell-permeable analogue of cAMP (pCPT-cAMP) (Fig. 1A and Supplementary Fig. S1A) selectively induced PHD3 mRNA expression to a greater extent than hypoxia-induced expression of *Phd3* mRNA (Fig. 1A). PHD3 mRNA and protein expression was induced by pCPT-cAMP in a time-dependent manner, and peaked at 180 min (Fig. 1B) and 360 min (Fig. 1C), respectively. *Phd3* induction by pCPT-cAMP in hepatocytes was inhibited by the PKA inhibitor H89 (Fig. 1D) but not by insulin (Supplementary Fig. S1B). Hepatic expression of *Phd3* transcript was ~2-fold higher in two mouse models of obesity-associated type 2 diabetes—i.e. normal mice fed a high-fat diet (Fig. 1E) and *db*/*db* mice (Fig. 1F)—as compared to their respective controls. This upregulation of the *Phd3* gene enhanced level of the protein in *db/db* mice (Fig. 1G). Taken together, these data indicate that *Phd3* gene expression in hepatocytes is regulated by glucagon–cAMP–PKA signalling.

### *Phd3* gene induction is mediated through a GCN5–CITED2–PKA signalling module

**Figure 2:**
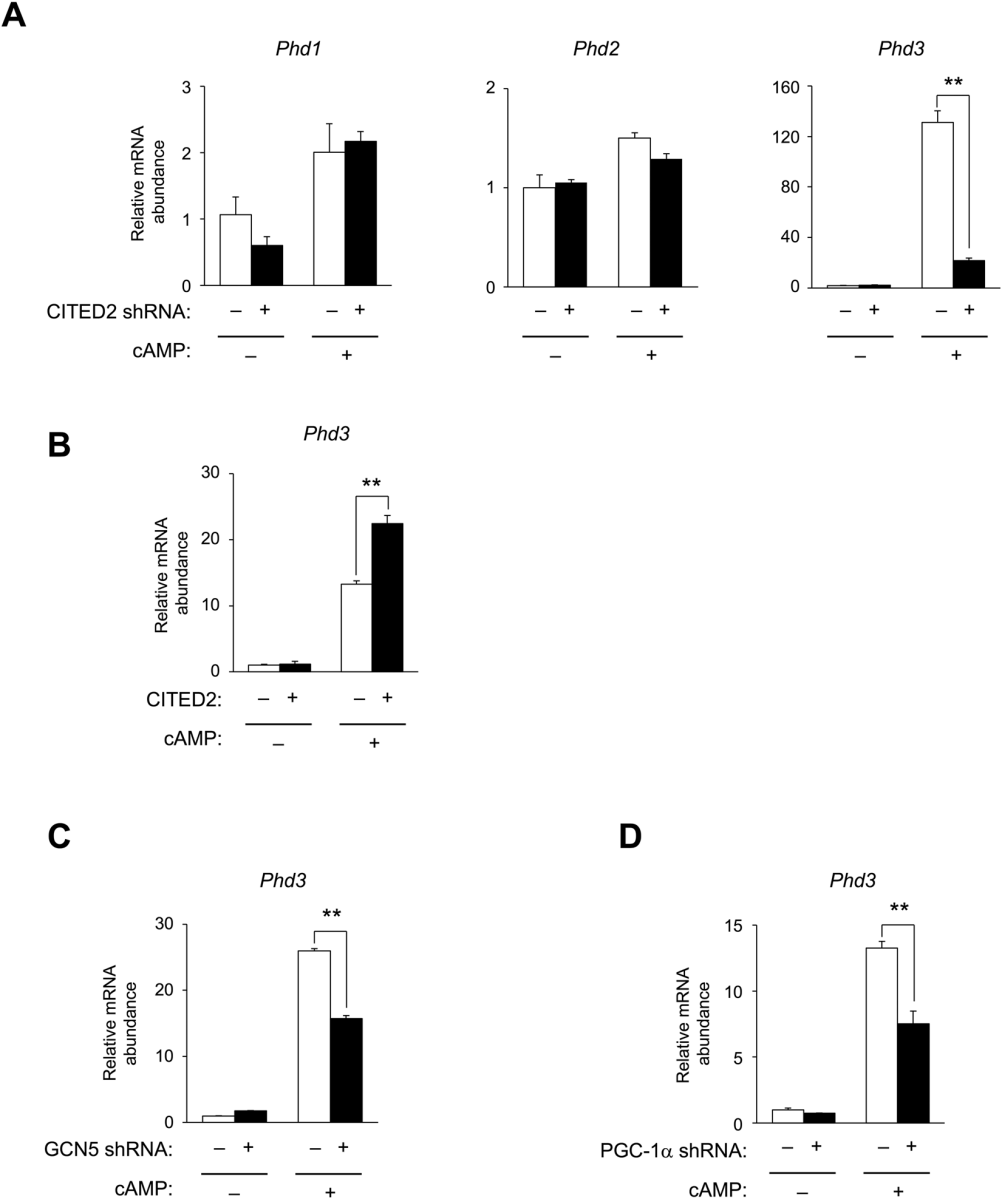
GCN5–CITED2–PKA signalling module mediates cAMP induction of *PHD3* gene expression. (**A**) Effects of shRNA-mediated CITED2 knockdown on mRNA expression of three PHD isoforms in primary mouse hepatocytes with or without exposure to pCPT-cAMP (100 μM, 6 h). (**B**) Effects of ectopic CITED2 expression on *Phd3* gene expression in primary mouse hepatocytes with or without exposure to pCPT-cAMP (100 μM, 6 h). (**C**,**D**) Effects of shRNA-mediated GCN5 (**C**) or PGC-1α (**D**) knockdown on *Phd3* gene expression in primary mouse hepatocytes with or without exposure to pCPT-cAMP (100 μM, 6 h). Data are shown as mean ± SEM (n = 3) and are representative of at least two independent experiments. Differences between groups were evaluated by ANOVA followed by Bonferroni’s post hoc test. **P < 0.01 vs. indicated groups. Adenoviral vectors encoding CITED2 and shRNAs targeting CITED2, GCN5, and PGC-1α were used for experiments.

We next investigated whether cAMP-dependent induction of the *Phd3* gene is mediated through a GCN5–CITED2–PKA signalling module in mouse hepatocytes. Depletion of CITED2 by ~95% using a short hairpin (sh)RNA adenovirus (Supplementary Fig. S2A) decreased cAMP-dependent induction of the *Phd3* gene by ~85% (Fig. 2A), whereas CITED2 overexpression nearly doubled the induction (Fig. 2B and Supplementary Fig. S2B). In contrast, CITED2 depletion did not affect the expression of *Phd*1 and *Phd2* genes (Fig. 2A). ShRNA-mediated knockdown of GCN5 (Fig. 2C and Supplementary Fig. S2C) or PGC-1α (*Ppargc1a*) (Fig. 2D and Supplementary Fig. S2D) also decreased cAMP induction of the *Phd3* gene by >40%. Disruption of this signalling module by depletion of CITED2 or GCN5 or PGC-1α—a downstream effector of this module—suppressed *Phd3* gene induction. These data indicate that *Phd3* gene expression is induced through a GCN5–CITED2–PKA signalling module.

### PHD3 is required for cAMP-dependent induction of gluconeogenesis

**Figure 3:**
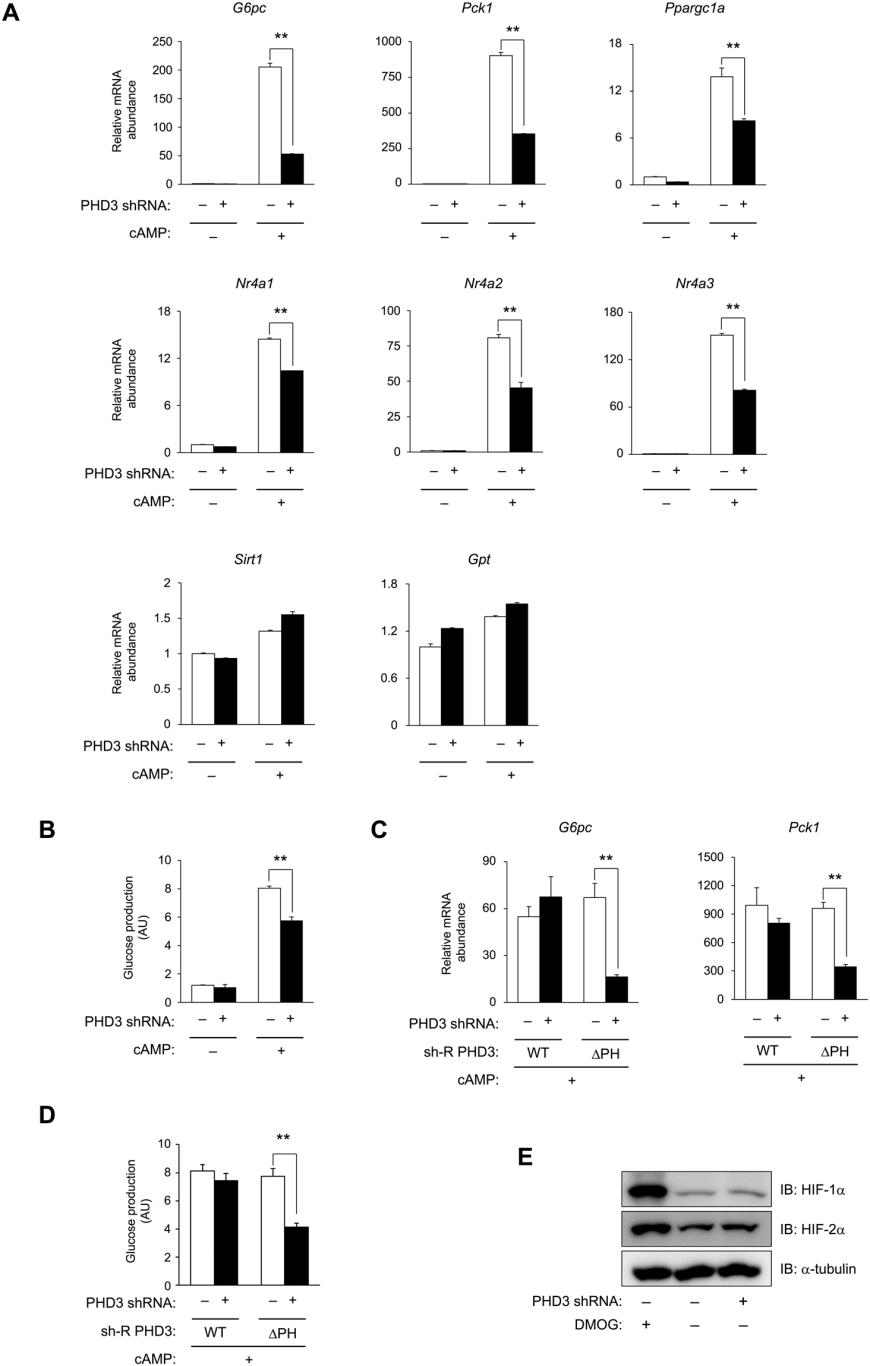
PHD3 is required for cAMP-dependent induction of gluconeogenesis. (**A**,**B**) Effects of shRNA-mediated PHD3 knockdown on gluconeogenic gene expression (**A**) and glucose production (**B**) in primary mouse hepatocytes with or without exposure to pCPT-cAMP for 6 and 22 h, respectively. (**C**,**D**) Effects of ectopic expression of shRNA-resistant PHD3(WT) or PHD3(ΔPH) on gluconeogenic gene expression (**C**) and glucose production (**D**) in primary mouse hepatocytes with or without shRNA-mediated knockdown of PHD3 in the presence of pCPT-cAMP (100 μM, 6 h). (**E**) Effects of shRNA-mediated PHD3 knockdown on HIF-1α and -2α protein levels in hepatocytes with or without exposure to DMOG (1 mM, 4 h). α-Tubulin served as the loading control for immunoblotting. Complete immunoblots are presented in Supplementary Fig. S7. Quantitative data are shown as mean ± SEM (n = 3) and are representative of at least two independent experiments. Differences between groups were evaluated by ANOVA followed by Bonferroni’s post hoc test. **P < 0.01 vs. indicated groups. Adenoviral vectors encoding PHD3 shRNA, sh-R PHD3(WT), or sh-R PHD3(ΔPH) were used for experiments.

It was previously reported that the GCN5–CITED2–PKA signalling module plays a critical role in the activation of a gluconeogenic program^8^. We examined the role of PHD3 in this process and found that PHD3 depletion by ~95% using an shRNA adenovirus (Supplementary Fig. S3A) decreased cAMP-induced expression of the gluconeogenic genes *G6pc* and *Pck1* by ~75% (Fig. 3A). This was accompanied by the impairment in the induction of key gluconeogenic transcriptional effectors targeted by CREB, such as *Ppargc1a*^27^ and *nuclear receptor subfamily 4 group A members 1–3* (also known as *Nur77*, *Nurr1*, and *Nor1*, respectively)^28^ (Fig. 3A). In contrast, PHD3 depletion did not affect the expression of *Sirt1* and *glutamic pyruvic transaminase* (*Gpt*) in the presence or absence of pCPT-cAMP (Fig. 3A). Similar inhibition of gluconeogenic gene induction was observed in hepatocytes isolated from liver-specific *Phd3* knockout mice (Supplementary Fig. S3B) or from mice homozygous for a floxed *Phd3* allele expressing Cre recombinase specifically in the liver (Supplementary Fig. S3C). Consistent with these results, PHD3 depletion prevented the cAMP-dependent release of glucose by hepatocytes into the culture medium (Fig. 3B), indicating that these changes in gene expression attenuate gluconeogenesis.

We next tested whether the prolyl hydroxylase activity of PHD3 is required for cAMP induction of gluconeogenic genes and glucose production using shRNA-resistant wild-type PHD3 [sh-R PHD3(WT)] and shRNA-resistant PHD3 lacking prolyl hydroxylase activity [sh-R PHD3(ΔPH)] in hepatocytes with shRNA-mediated depletion of PHD3. Expression of sh-R PHD3(WT) restored cAMP induction of *G6pc* and *Pck1* genes, whereas sh-R PHD3(ΔPH) had only a partial effect (Fig. 3C and Supplementary Fig. S3D). Consistent with the changes in gluconeogenic gene expression, expression of sh-R PHD3(WT) restored cAMP-induced glucose production, whereas sh-R PHD3(ΔPH) had only a partial effect (Fig. 3D). It is worth noting that HIF-1α and -2α proteins were induced in primary hepatocytes treated with dimethyloxaloylglycine (DMOG)—a cell-permeable inhibitor of HIF hydroxylases including PHDs—but not in *Phd3*-deficient hepatocytes, suggesting that inhibition of gluconeogenesis by PHD3 depletion is independent of HIFs (Fig. 3E and Supplementary Fig. S3E). Collectively, these data indicate that PHD3—specifically, its prolyl hydroxylase activity—is required for cAMP-dependent induction of gluconeogenesis in a HIF-independent manner.

### PHD3 interacts with CITED2 and GCN5 to regulate PGC-1α-induced gluconeogenesis

**Figure 4:**
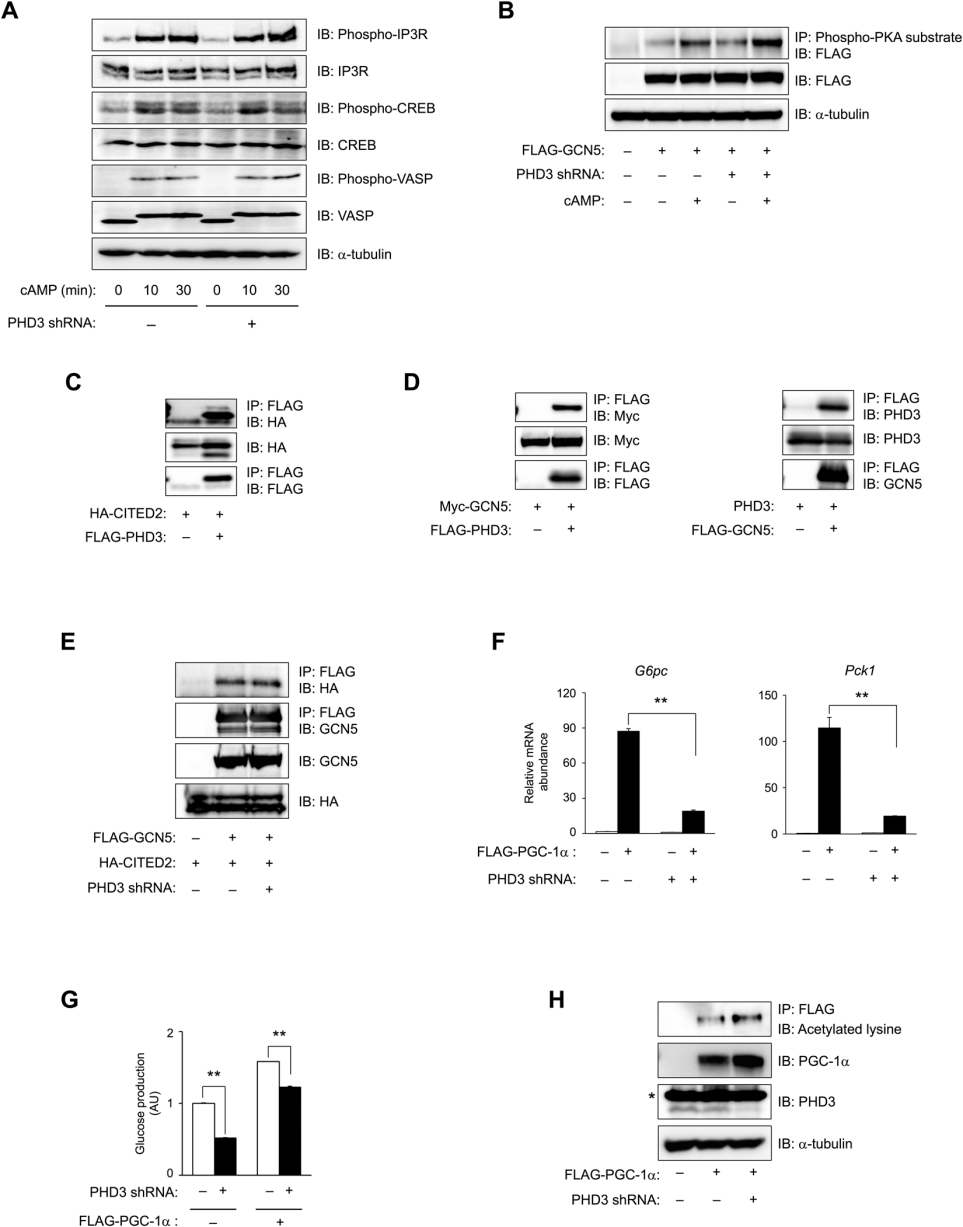
PHD3 interacts with CITED2 and GCN5 and regulates PGC-1α-induced gluconeogenesis. (**A**) Effects of PHD3 depletion on phosphorylation of various PKA substrates induced by pCPT-cAMP (100 μM for 0, 10, and 30 min) in primary hepatocytes. (**B**) Mouse hepatocytes with or without FLAG-tagged GCN5 expression and with or without PHD3 knockdown were exposed to 100 μM pCPT-cAMP for 30 min or left untreated, and then subjected to immunoprecipitation with antibodies against phosphorylated PKA substrates followed by immunoblot analysis with anti-DYKDDDDK antibody. (**C**,**D**) Immunoprecipitation and immunoblot analyses of the interaction between FLAG-PHD3 and HA-CITED2 (**C**), FLAG-PHD3 and Myc-GCN5, and PHD3 and FLAG-GCN5 (**D**) in AD-293 cells. (**E**) Effect of PHD3 depletion on the interaction between FLAG-GCN5 and HA-CITED2 in AML12 cells. (**F**,**G**) Effects of PHD3 depletion on PGC-1α-induced gluconeogenic gene expression (**F**) and glucose production (**G**) in primary mouse hepatocytes with or without FLAG-PGC-1α expression in the absence of pCPT-cAMP. (**H**) Immunoprecipitation and immunoblot analyses of PGC-1α acetylation in primary hepatocytes expressing FLAG–PGC-1α with or without PHD3 depletion. α-Tubulin served as the loading control for immunoblotting. *Non-specific. Complete immunoblots are presented in Supplementary Fig. S7. All quantitative data are shown as mean ± SEM (n = 3 (**F**,**G**)) and are representative of at least two independent experiments. Differences between groups were evaluated by ANOVA followed by Bonferroni’s post hoc test (**F**,**G**). **P < 0.01 vs. indicated groups. Adenoviral vectors encoding PHD3 shRNA, control shRNA, FLAG-GCN5, HA-CITED2, and FLAG-PGC-1α were used for experiments.

PKA is activated by cAMP and induces gluconeogenesis through phosphorylation of GCN5 in the GCN5–CITED2–PKA signalling module^8^ and CREB^27,29^ and inositol 1,4,5-trisphosphate receptor (IP3R)^30^ in hepatocytes. PKA also mediates actin cytoskeleton remodelling by phosphorylating vasodilator-stimulated phosphoprotein (VASP). To clarify the mechanism by which PHD3 regulates cAMP-induced gluconeogenesis, we examined whether phosphorylation of these PKA substrates is affected by PHD3 depletion. ShRNA-mediated knockdown of PHD3 in hepatocytes did not affect the phosphorylation of IP3R, CREB, and VASP by pCPT-cAMP (Fig. 4A) as well as PKA-dependent phosphorylation of GCN5 (Fig. 4B), as determined by immunoblotting. Co-immunoprecipitation analysis in AD-293 cells revealed that PHD3 interacts with both CITED2 (Fig. 4C) and GCN5 (Fig. 4D). The CITED2–GCN5 interaction is critical for PGC-1α coactivation^22^; in AML12 cells expressing epitope-tagged CITED2 and GCN5, PHD3 knockdown did not alter the amount of CITED2 co-immunoprecipitated with GCN5, indicating that the CITED2–GCN5 interaction was unaffected (Fig. 4E). These data suggest that PHD3 interacts with GCN5 and CITED2 in the GCN5–CITED2–PKA signalling module without affecting module assembly or PKA-dependent phosphorylation of GCN5.

We next investigated whether PHD3 regulates PGC-1α—another gluconeogenic effector downstream of this module—by examining the effect of PHD3 depletion on PGC-1α-induced gluconeogenic gene expression and glucose production in primary hepatocytes. Ectopic expression of PGC-1α in the absence of pCPT-cAMP increased *G6pc* and *Pck1* expression ~90 and ~120 fold, respectively; however, PHD3 knockdown decreased induction by 80% (Fig. 4F and Supplementary Fig. S4A). Consistent with the changes in gluconeogenic gene expression, PHD3 knockdown decreased PGC-1α-induced glucose production (Fig. 4G). As PGC-1α co-activation is negatively regulated by GCN5-dependent acetylation^10^, we assessed the acetylation status of PGC-1α in this context and found that it was unaffected by PHD3 depletion (Fig. 4H). Taken together, these results indicate that PHD3 interacts with CITED2 and GCN5 and regulates PGC-1α-induced gluconeogenesis in a proline hydroxylase-dependent manner independent of GCN5-mediated acetylation of PGC-1α.

### PHD3 depletion suppresses insulin signalling independent of prolyl hydroxylase

**Figure 5:**
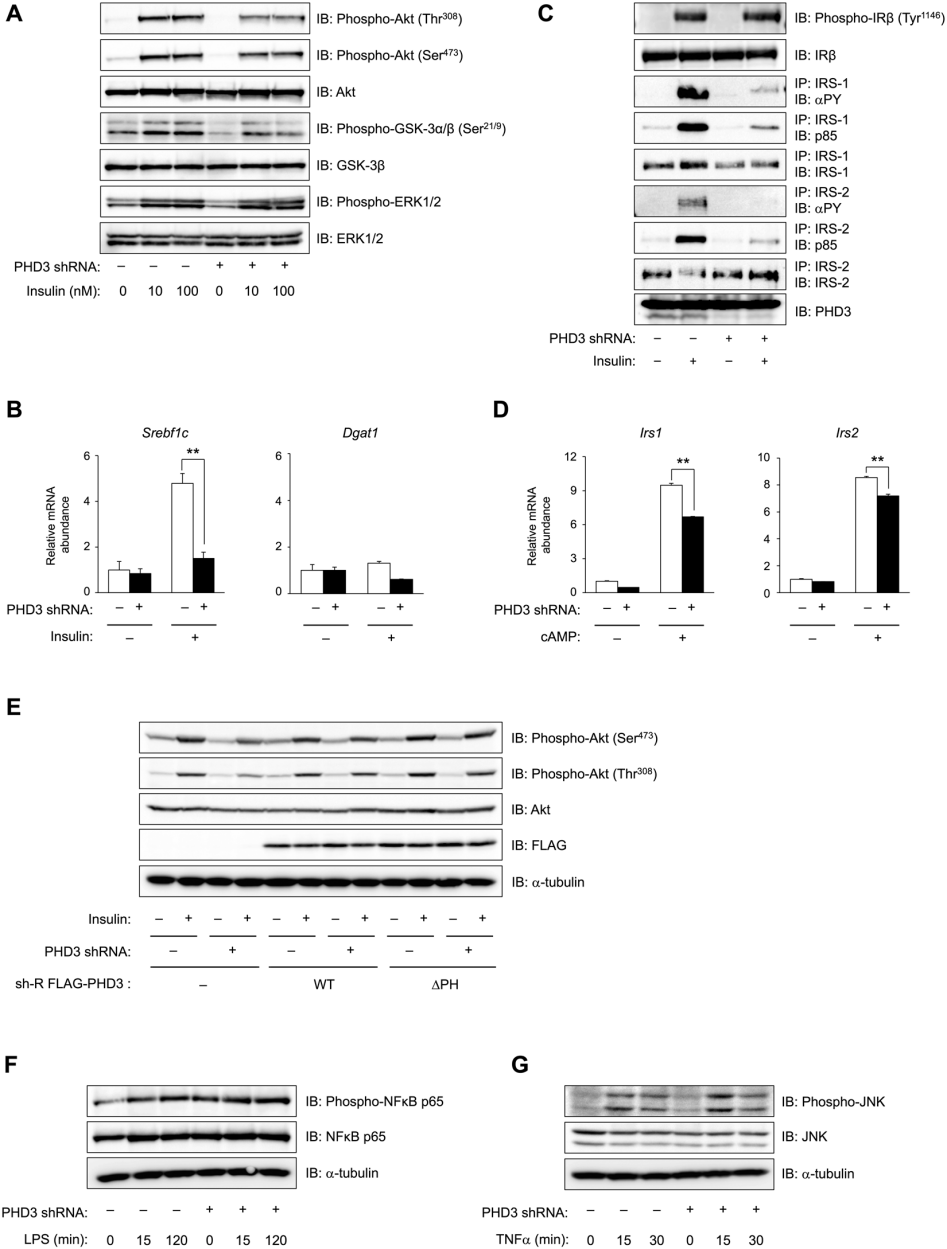
PHD3 depletion impairs insulin signalling associated with NF-κB and JNK activation. (**A**) Effects of PHD3 depletion on insulin-induced (10 or 100 nM, 10 min) phosphorylation of Akt at Thr^308^ and Ser^473^, GSK-3α/β at Ser^21/9^, and extracellular signal-regulated kinase (ERK)1/2 at Thr^202^/Tyr^204^, and total protein levels of Akt, GSK-3β and ERK1/2 in mouse hepatocytes, as determined by immunoblotting. (**B**) Effects of shRNA-mediated PHD3 knockdown on *Srebf1c* and *Dgat1* mRNA expression in primary mouse hepatocytes with or without exposure to 10 nM insulin for 6 h. (**C**) Mouse hepatocytes with or without PHD3 depletion were exposed to 10 nM insulin for 1 min or left untreated, and then subjected to immunoblot analysis with an antibody specific to Tyr1146-phosphorylated IR β subunit (IRβ). Cells were also subjected to immunoprecipitation with antibodies against IRS-1 or -2, followed by immunoblot analysis with antibodies against phosphorylated tyrosine (αPY), PI3K p85 subunit, or IRS-1 or -2. (**D**) Effects of PHD3 depletion on *Irs1* and *Irs2* mRNA levels in primary mouse hepatocytes with or without exposure to 100 μM pCPT-cAMP for 6 h. (**E**) Immunoblot analysis of the effects of enforced expression of shRNA-resistant PHD3(WT) or PHD3(ΔPH) on insulin-induced (10 nM, 10 min) phosphorylation of Akt at Thr^308^ and Ser^473^ in primary mouse hepatocytes with or without PHD3 depletion. (**F**,**G**) Effects of PHD3 depletion on phosphorylation of NF-κB p65 subunit at Ser^536^ (**F**) and JNK at Thr^183^/Tyr^185^ (**G**) in mouse hepatocytes with or without exposure to LPS (100 ng/ml) (**F**) or TNF-α (20 ng/ml) (**G**) for indicated times. α-Tubulin served as the loading control for immunoblotting. Complete immunoblots are presented in Supplementary Fig. S7. Quantitative data are shown as mean ± SEM (n = 3 (**B**,**D**)) and are representative of at least two independent experiments. Differences between groups were evaluated by ANOVA followed by Bonferroni’s post hoc test (**B**,**D**). **P < 0.01 vs. indicated groups. Adenoviral vectors encoding PHD3 shRNA, sh-R PHD3(WT), or sh-R PHD3(ΔPH) were used for experiments.

Acute depletion of hepatic PHD3 in mice has been shown to improve whole body insulin sensitivity and diabetes by stabilising HIF-2α, thereby enhancing *Irs2* gene transcription and insulin-stimulated Akt activation and decreasing the expression of gluconeogenic genes such as *G6pc*, *Pck1*, and *Ppargc1a* in the liver^31^. To determine whether this can be recapitulated *ex vivo*, we examined the effect of PHD3 knockdown on insulin signalling in hepatocytes. Unexpectedly, insulin-stimulated phosphorylation of Akt at Thr^308^ and Ser^473^ and of glycogen synthase kinase (GSK)-3α/β at Ser^21/9^, but not of extracellular signal-regulated kinase 1/2 at Thr^202^/Tyr^204^, was attenuated in the absence of PHD3 as compared to control cells (Fig. 5A). Consistent with these findings, insulin-stimulated induction of *sterol regulatory element-binding protein* (*Srebp*)*1c*—a key transcription factor for de novo lipogenesis^32^ whose expression is mediated by the phosphoinositide 3-kinase (PI3K) effectors Akt^33^ and PKCλ^34^—was inhibited by PHD3 knockdown (Fig. 5B). However, the mRNA level of *diacylglycerol O-acyltransferase* (*Dgat*)*1*—encoding an enzyme that catalyses the final step in triacylglycerol synthesis—was unaffected (Fig. 5B). We also examined the effect of PHD3 knockdown on the insulin signalling components upstream of Akt and found that it did not affect insulin-induced Tyr^1146^ phosphorylation and protein level of the IR β subunit (Fig. 5C). IRS-1 and-2 tyrosine phosphorylation and association with the PI3K p85 subunit induced by insulin were abolished by loss of PHD3, although the protein levels were unaffected (Fig. 5C). Similar changes in insulin signalling were observed in PHD3-deficient hepatocytes (Supplementary Figs S5A and S5B). It should be noted that unlike in the *in vivo* setting^31^, transcript levels of *Irs2* and *Irs1* were not upregulated following exposure to pCPT-cAMP but were instead reduced in the absence of PHD3 (Fig. 5D). We also examined whether the prolyl hydroxylase activity of PHD3 is required for insulin signal transduction in PHD3-depleted hepatocytes expressing sh-R PHD3(WT) or sh-R PHD3(ΔPH). Ectopic expression of these constructs did not affect insulin signalling in control hepatocytes (Supplementary Fig. S5C); however, in cells lacking PHD3, insulin-stimulated Akt phosphorylation was restored (Fig. 5E). These data suggest that PHD3 plays a critical role in maintaining insulin signalling in a prolyl hydroxylase-independent manner, and that loss of PHD3 impairs insulin signalling at the level of IRS tyrosine phosphorylation.

### PHD3 depletion activates IKK- and JNK-mediated stress signalling pathways

Insulin signalling is blocked by inhibitory serine/threonine phosphorylation of IRS proteins, which is directly mediated by JNK^19^ and IKKβ^20^ activated by stress and cytokine (e.g. tumour necrosis factor [TNF]-α) signalling. This inhibitory phosphorylation is also mediated by mTOR and p70 S6 kinase^35^ as a counter-regulatory loop of insulin signalling. On the other hand, IKKβ activates NF-κB, a master regulator of the stress response, which drives the expression of proinflammatory cytokines such as TNF-α and interleukin (IL)-6 and of enzymes such as inducible nitric oxide synthase (iNOS)^36^. We investigated whether PHD3 depletion affects the stress, cytokines, and insulin signalling cascades in hepatocytes and found that IKK-dependent phosphorylation of the NF-κB p65 subunit at Ser^534^ (the murine homologue of human Ser^536^) was enhanced under both basal and lipopolysaccharide (LPS)-stimulated conditions (Fig. 5F and Supplementary Fig. S5D). PHD3 knockdown also increased the phosphorylation of JNK at Thr^183^ and Tyr^185^ under basal and TNF-α-stimulated conditions (Fig. 5G) but reduced mTOR complex 1-dependent phosphorylation of p70 S6 kinase at Thr^389^, Thr^421^, and Ser^424^ (ref.^37^) under insulin stimulation (Supplementary Fig. S5E), consistent with impaired insulin signalling. Thus, PHD3 depletion activates IKK and JNK stress kinases, which suppress insulin signalling at the level of IRS.

### PHD3 represses NF-κB-induced proinflammatory gene expression in a prolyl hydroxylase-independent manner

**Figure 6:**
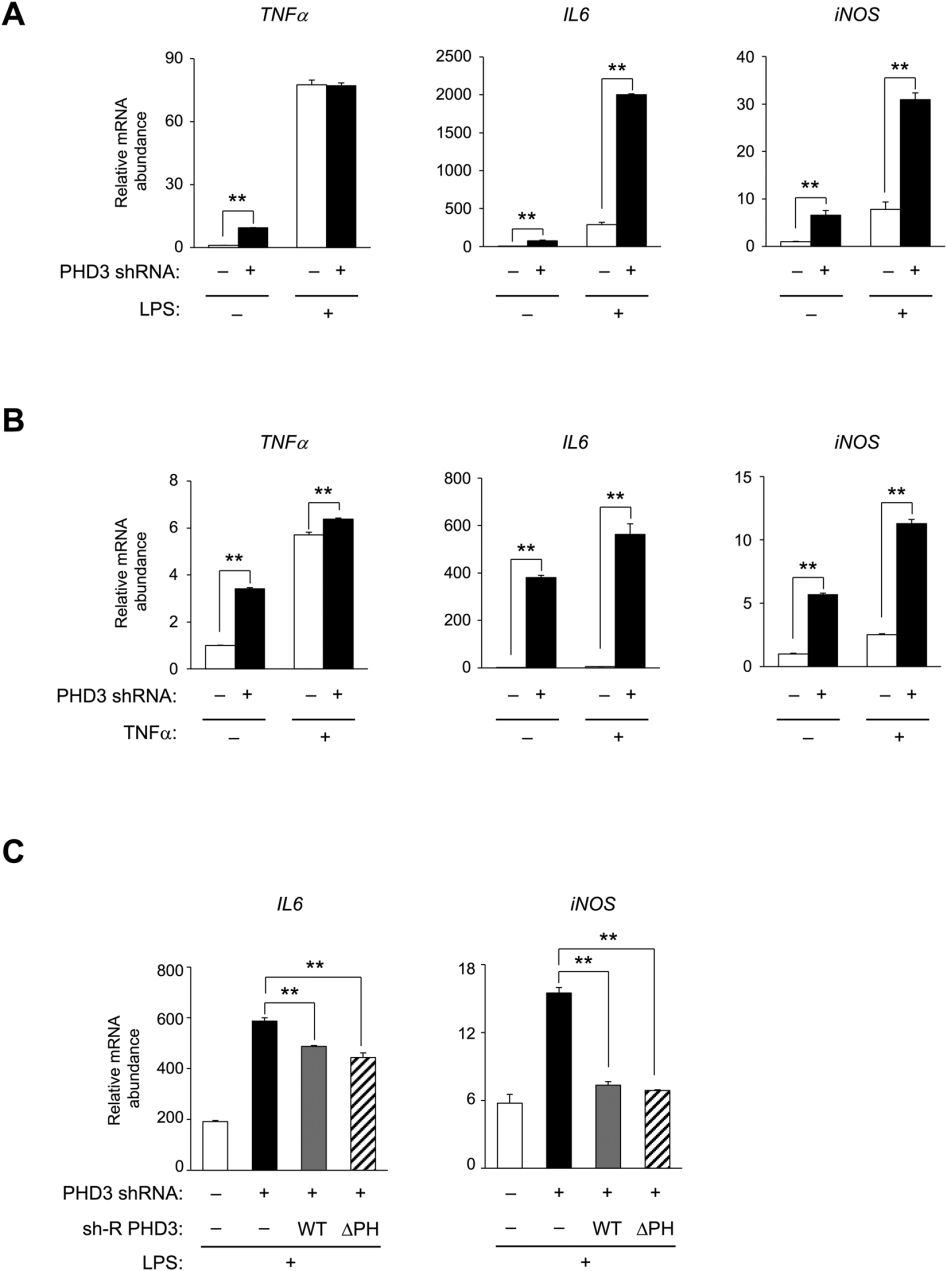
PHD3 represses NF-κB-mediated transcription of proinflammatory genes independent of prolyl hydroxylase activity. (**A**,**B**) Effects of shRNA-mediated PHD3 knockdown on proinflammatory gene expression in primary mouse hepatocytes with or without exposure to 100 ng/ml LPS (**A**) or 20 ng/ml TNF-α (**B**) for 2 h, as detected by qRT-PCR. (**C**) Effects of ectopic expression of shRNA-resistant PHD3(WT) or PHD3(ΔPH) on *IL-6* and *iNOS* gene expression in PHD3-depleted primary mouse hepatocytes in the presence of LPS (100 ng/ml, 2 h), as detected by qRT-PCR. Quantitative data are shown as mean ± SEM (n = 3) and are representative of at least two independent experiments. Differences between groups were evaluated by ANOVA followed by Bonferroni’s post hoc test. **P < 0.01 vs. indicated groups. Adenoviral vectors encoding PHD3 shRNA, sh-R PHD3(WT), or sh-R PHD3(ΔPH) were used for experiments.

To clarify whether NF-κB is activated by PHD3 depletion, we examined the expression of target proinflammatory cytokines and enzymes and found that *TNF-α*, *IL-6*, and *iNOS* mRNA levels were increased ~10, ~380, and ~7 fold, respectively, in untreated PHD3-depleted hepatocytes (Fig. 6A,B). LPS (Fig. 6A)- and TNF-α (Fig. 6B)-induced expression of *IL-6* and *iNOS* and TNF-α-induced expression of *TNF-α* (Fig. 6B) were markedly increased in PHD3-depleted hepatocytes. Basal and LPS-induced expression of *TNF-α*, *IL-6*, and *iNOS* was also upregulated in PHD3 knockout hepatocytes (Supplementary Fig. S6A). Identical changes in basal levels of these genes were observed in hepatocytes homozygous for a floxed *Phd3* allele removed by adenoviral Cre-mediated recombination (Supplementary Fig. S6B). We examined whether the prolyl hydroxylase activity of PHD3 is required for this effect, given that loss of PHD3 expression induces proinflammatory NF-κB target genes. The enhancement of LPS-stimulated upregulation of *IL-6* and *iNOS* induced by PHD3 knockdown was abolished by expression of either sh-R PHD3(WT) or sh-R PHD3(ΔPH) (Fig. 6C and Supplementary Fig. S6C). These data suggest that PHD3 inhibits NF-κB and the expression of its downstream target genes in a prolyl hydroxylase-independent manner.

### PHD3 depletion enhances IL-6–signal transducer and activator of transcription (STAT)3 signalling and a branch of the unfolded protein response (UPR) independent of prolyl hydroxylase

**Figure 7:**
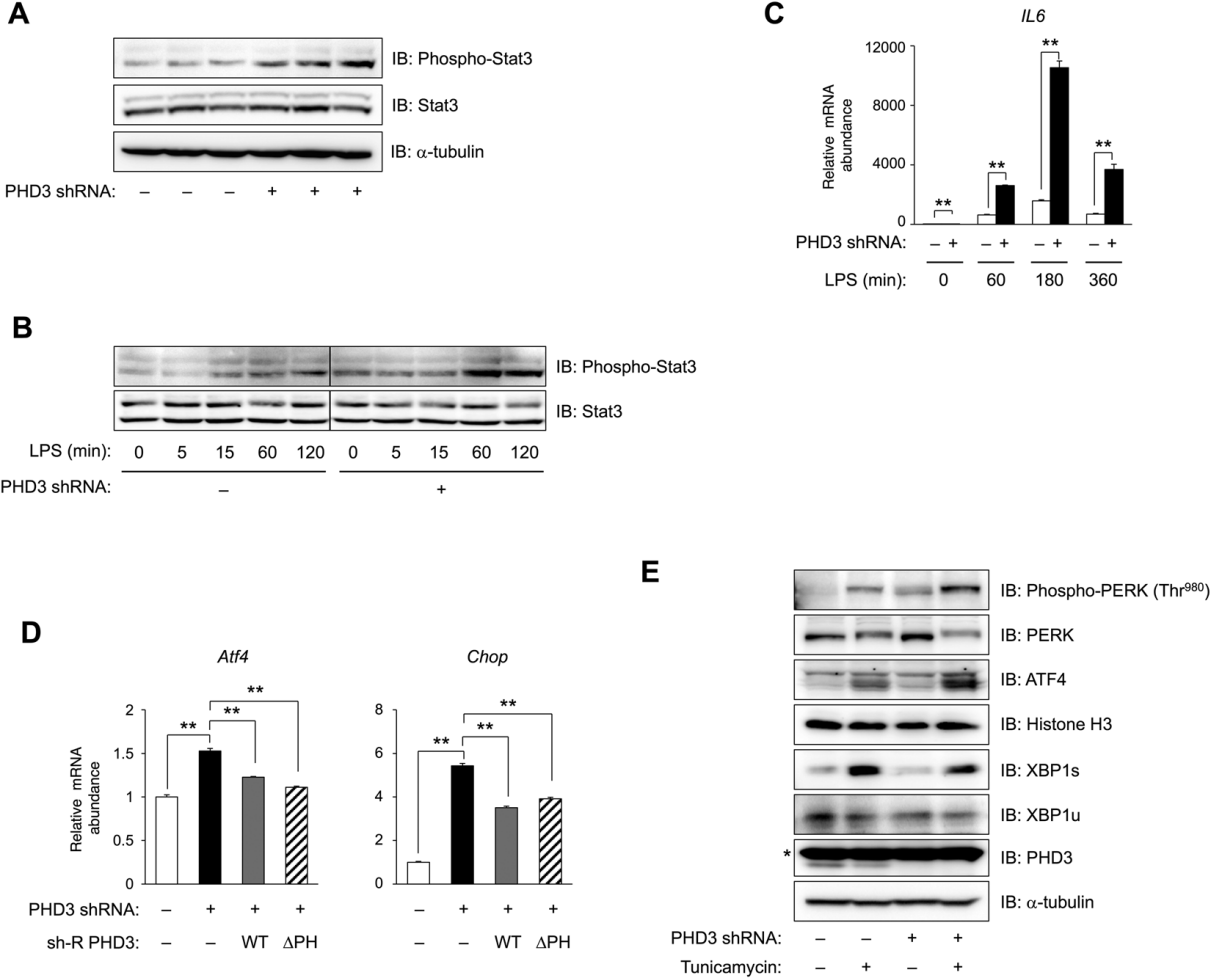
PHD3 depletion potentiates IL-6–STAT3 signalling and the PERK–ATF4 branch of the UPR^ER^ pathway independent of prolyl hydroxylase activity. (**A**,**B**) Effects of shRNA-mediated PHD3 knockdown on Tyr^705^ phosphorylation and STAT3 protein level in mouse hepatocytes without (**A**) or with (**B**) exposure to 100 ng/ml LPS for indicated times, as detected by immunoblotting. The lines in (**B**) indicate the deletion of non-relevant bands from the blots. (**C**) Time course analysis of *IL-6* mRNA expression in mouse hepatocytes with or without PHD3 depletion exposed to 100 ng/ml LPS for indicated times. (**D**) Effects of ectopic expression of shRNA-resistant PHD3(WT) or PHD3(ΔPH) on *Atf4* and *Chop* gene expression induced by PHD3 depletion in primary mouse hepatocytes, as detected by qRT-PCR. (**E**) Effect of PHD3 depletion on Thr^980^ phosphorylation of PERK and total PERK, XBP1s, XBP1u, PHD3, and α-tubulin levels in whole cell lysates, and total amount of nuclear ATF4 and histone H3 in AML12 cells with or without exposure to 5 μg/ml tunicamycin for 24 h, as determined by immunoblotting. Histone H3 and α-tubulin served as loading controls for immunoblot analyses of the nuclear fraction and whole cell lysates, respectively. *Non-specific. Complete immunoblots are presented in Supplementary Fig. S7. Quantitative data are shown as mean ± SEM (n = 3 (**C**,**D**)) and are representative of at least two independent experiments. Differences between groups were evaluated by ANOVA followed by Bonferroni’s post hoc test. **P < 0.01 vs. indicated groups. Adenoviral vectors encoding PHD3 shRNA, sh-R PHD3(WT), or sh-R PHD3(ΔPH) were used for experiments.

The IL-6–STAT3 axis has been shown to suppress gluconeogenic gene expression^38,39^. Given our observations that loss of PHD3 potentiated TNF-α–JNK and TNF-α–IKK–NF-κB axes with concurrent upregulation of *IL-6* while inhibiting insulin signalling, we investigated whether PHD3 depletion-induced upregulation of *IL-6* transcription activates IL-6–STAT3 signalling. STAT3 phosphorylation at Tyr^705^ by Janus kinase family members was enhanced in PHD3-depleted hepatocytes under basal conditions and in the presence of LPS (Fig. 7A,B). *IL-6* expression (Fig. 7C) was enhanced in PHD3-depleted but not in control hepatocytes under both conditions, suggesting that PHD3 knockdown stimulates IL-6 production via NF-κB activation.

NF-κB is activated in obesity—a state of chronic overnutrition^40^—by persistent endoplasmic reticulum (ER) stress: activation of the protein kinase RNA-like ER kinase (PERK)–eukaryotic translation initiation factor (eIF)2α pathway suppresses IκB translation^41^, while activated inositol-requiring protein (IRE)1α interacts with TNF receptor-associated factor 2 to activate IKK^42^ as well as JNK^43^, leading to NF-κB activation. On the other hand, a recent study reported that chronic inflammation in obese mice compromises the IRE1α–X-box-binding protein (XBP)-1 branch of the UPR in the ER (UPR^ER^). The activity of iNOS, an NF-κB-regulated inflammatory mediator, is upregulated in the liver of obese mice, which causes S-nitrosylation of IRE1α and impairment of XBP-1 splicing activity; this maladaptive UPR leads to dysregulation of insulin signalling^44^. Since persistent inflammation and ER stress are both induced in the UPR^ER^ and reduce sensitivity to insulin, we investigated whether PHD3 affects the UPR^ER^ pathway. PHD3 knockdown increased mRNA (Fig. 7D) and protein (Fig. 7E) levels of activating transcription factor (ATF)4 and the transcript level of its target gene CCAAT/enhancer-binding protein homologous protein (*Chop*) (Fig. 7D). Since PERK activation upon ER stress leads to upregulation of ATF4, we evaluated PERK activity based on its phosphorylation at Thr^980^. Loss of PHD3 enhanced PERK Thr^980^ phosphorylation in the absence or presence of tunicamycin, an inducer of ER stress (Fig. 7E). In contrast, the level of the spliced form of XBP1 (XBP1s), which is an active transcription factor, was decreased by PHD3 depletion (Fig. 7E), suggesting that the PERK–ATF4 branch of the UPR^ER^ is specifically activated in the absence of PHD3.

Finally, we tested whether the prolyl hydroxylase activity of PHD3 is required to suppress *Atf4* and *Chop* gene transcription. The induction of both genes in PHD3-depleted hepatocytes was partly reversed by expressing either sh-R PHD3(WT) or sh-R PHD3(ΔPH) (Fig. 7D). Thus, in the absence of PHD3, the stress signalling pathway is induced via NF-κB activation in a prolyl hydroxylase-independent manner, indicating that PHD3 is a negative regulator of this process.

## Discussion

**Figure 8:**
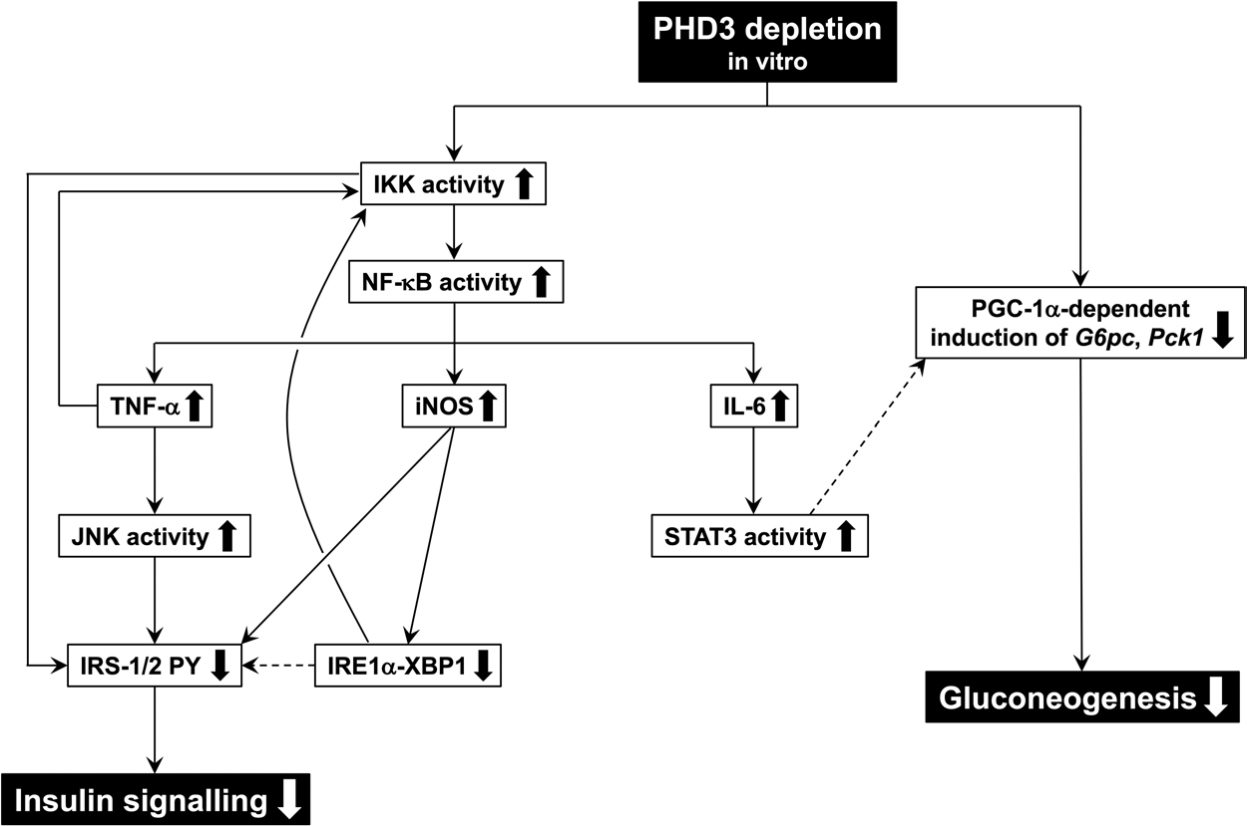
Proposed mechanism by which PHD3 depletion leads to decreased gluconeogenesis, impaired insulin signalling, and enhanced stress signalling in hepatocytes. PY: phosphorylation of tyrosine residues.

Glucagon signalling during fasting induces diverse metabolic responses in hepatocytes to meet fasting energy demands and to prepare for postprandial nutrient processing. Here we show that PHD3—whose expression is upregulated by glucagon–cAMP signalling through the GCN5–CITED2–PKA signalling module—is a critical component of cAMP-induced gluconeogenesis and insulin signal transduction, two key processes for maintaining glucose homeostasis in both fasting and postprandial states. Using loss- and gain-of-function approaches, we demonstrated that in PHD3-depleted hepatocytes, insulin signalling is compromised by activation of various stress signalling pathways independent of the prolyl hydroxylase activity of PHD3, whereas impairment of PGC-1α-dependent gluconeogenesis is largely dependent on this activity but independent of stress signalling (Fig. 8). PHD3 depletion activates the IKK–NF-κB pathway, leading to the production of proinflammatory cytokines such as TNF-α and IL-6 as well as NO, with consequent activation of stress signalling. These results highlight the inhibitory role of PHD3 in these pathways. Activation of IKK^20,45^ and JNK^19^ as well as increased cellular NO levels through upregulation of *iNOS* ^46^ was shown to dysregulate insulin signalling at the level of IRS in PHD3-depleted hepatocytes. Given that the induction of proinflammatory gene expression and compromised insulin signalling in hepatocytes is independent of PHD3 activity, activation of JNK and IKK and induction of iNOS may impair insulin signalling.

PHD3 depletion may activate IKK by both TNF-α-dependent and -independent mechanisms. In certain cell types, TNF-α produced through activation of IKK–NF-κB signalling is known to further enhance this pathway^47^. This autocrine positive feedback loop may function in hepatocytes in the absence of PHD3. On the other hand, PHD3-induced suppression of the IKK complex may be a TNF-α-independent mechanism. The IKK complex consists of two catalytic subunits (IKKα and IKKβ) and one regulatory scaffold subunit (IKKγ, also known as NF-κB essential modulator)^48^. Phosphorylation of IKKβ^49^ and ubiquitination of IKKγ^50^ induced by various stress stimuli or receptor-mediated signalling (e.g. TNF and Toll-like receptors) are essential for IKK and NF-κB activation. In non-hepatic cells, PHD3 binds to and inhibits the phosphorylation of IKKβ^51^ and ubiquitination of IKKγ^52^ induced by TNF-α, leading to activation of IKK–NF-κB signalling. These inhibitory effects of PHD3 are independent of its hydroxylase activity and may therefore be relevant in hepatocytes. PHD3 depletion may potentiate IKK activity through the suppression of such effects; our observation that loss of PHD3 in hepatocytes induced proinflammatory gene expression in the absence and presence of TNF-α or LPS supports this possibility. Whether IKK is activated through an TNF-α-dependent or -independent mechanism and the initiating signal in PHD3 depletion-induced activation of IKK remain to be determined in future studies.

In non-obese mice, hepatocyte-specific activation of NF-κB promotes gluconeogenesis through upregulation of genes encoding gluconeogenic enzymes, thereby perturbing insulin signalling^45^. On the other hand, suppressing the IKK–NF-κB pathway in obese and diabetic mice ameliorates diabetes, with a concomitant decrease in gluconeogenic gene expression and gluconegenesis^45,53^. These findings illustrate the molecular link between the hepatic IKK–NF-κB pathway, insulin signalling, and gluconeogenesis. Although PHD3 deficiency in hepatocytes was associated with IKK–NF-κB activation and impairment of insulin signalling, cAMP-dependent gluconeogenic gene expression was attenuated, suggesting that the inhibition of gluconeogenesis is caused by IKK–NF-κB-independent and proline hydroxylase-dependent mechanisms. PHD3 was shown to regulate gene expression through hydroxylation of various transcriptional regulators including the α-subunits of HIF-1/2^23,24^, ATF4^54^, thyroid hormone receptor-α^55^, and pyruvate kinase M2^56^. PHD3 co-immunoprecipitates with both CITED2 and GCN5, and presumably interacts with the GCN5–CITED2–PKA signalling module. However, loss of PHD3 function did not affect events related to this module that are critical for gluconeogenesis—i.e. the assembly of this module, PKA-dependent phosphorylation of GCN5, and GCN5-dependent acetylation of PGC-1α. PHD3 knockdown suppressed PGC-1α-dependent gluconeogenic gene induction, suggesting that it contributes to gluconeogenesis through hydroxylation of PGC-1α and downstream transcriptional machinery. We previously showed that the IL-6–STAT3 axis inhibits gluconeogenic gene expression^38,39^; it is also possible that in the absence of PHD3, IL-6 production induced by NF-κB suppresses gluconeogenesis through STAT3 activation.

Under conditions of chronic metabolic excess such as those seen in obesity, chronic low-grade inflammation and ER stress are thought to cooperatively induce hepatic insulin resistance^21,57^. In this setting, UPR^ER^ is aberrantly activated in association with PERK–eIF2α–ATF4 activation^58^, while the IRE1α–XBP-1 axis is suppressed^44^. It is worth noting that PHD3 deficiency caused similar changes in the UPR^ER^ concomitant with proinflammatory changes in hepatocytes. eIF2α is phosphorylated by PERK, suppresses IκB translation, and thereby activates IKK–NF-κB^41,59^, which may contribute to IKK–NF-κB activation in PHD3-depleted hepatocytes. PHD3 also suppresses ATF4 through hydroxylation-mediated degradation^54^. Increased ER stress and relief from translational suppression of ATF4 could explain the activation of the PERK–eIF2α–ATF4 axis in PHD3-depleted cells, although the underlying molecular mechanism remains to be elucidated. On the other hand, XBP1s induction in PHD3-deficient hepatocytes may be blocked by inactivation of IRE1α ribonuclease through iNOS-dependent S-nitrosylation^44^. Enhanced expression of PHD3 in the liver of *db/db* mice presumably resulting from a chronic excess of glucagon signalling may antagonise such stress signalling events.

Taniguchi *et al*. subacutely deleted PHD3 in the liver of mice homozygous for a floxed PHD3 allele by injecting adenovirus encoding Cre recombinase through the tail vein^31^. Such subacute depletion of PHD3 *in vivo* improved insulin sensitivity and glucose tolerance, and thereby ameliorated diabetes. In that study, PHD3 depletion stabilised HIF-2α, which in turn promoted transcription of *Irs2*, improved insulin signalling, and increased insulin sensitivity, although there was no information related to stress signalling pathways. In this study, we show that acute loss of PHD3 in primary cultured hepatocytes activates diverse stress signalling pathways without HIF-2α-dependent upregulation of *Irs2*. Further, using hepatocytes isolated from liver-specific PHD3 knockouts, we show that its chronic deletion fails to upregulate HIF-2α *in vitro*. The discrepancy in the effect of PHD3 loss of function on insulin signalling appear to be due to differences between *in vitro* vs. *in vivo* settings. Such discrepancies may be related to activation of stress signalling and induction of HIF-2α. The mechanism by which *in vitro* PHD3 depletion fails to induce HIF-2α expression remains to be elucidated.

In conclusion, our findings in hepatocytes indicate that PHD3 induced by cAMP regulates hormone-mediated glucose metabolism through suppression of stress signalling and optimisation of gluconeogenesis and insulin signalling. These effects of PHD3 are exerted via proline hydroxylase-dependent and -independent mechanisms. An *in vivo* acute loss-of-function analysis revealed that hepatic PHD3 regulates glucose metabolism in both isoform-specific and -overlapping manners^31^. Thus, PHD inhibition is a promising therapeutic intervention for a range of disorders including renal anaemia^60^, ischemic and inflammatory diseases^61^, and tissue injury^62^. It will be interesting to examine the role of PHD3 that we identified in various tissues in the context of chronic metabolic excess and stress, such as obesity and type 2 diabetes; clarifying this point along with the dependency on PHD3 enzymatic activity and isoform specificity can provide a basis for designing more effective therapeutic strategies that target PHD3.

## Methods

### Mice

Animal husbandry and experiments with mice were performed in accordance with the Regulation of Animal Experiments of the National Center for Global Health and Medicine (Tokyo, Japan), and were approved by the Institutional Animal Care and Use Committee of the National Center for Global Health and Medicine. Male C57BL/6J mice (CLEA Japan, Tokyo, Japan) and *db*/*db* (C57BLKS/J Iar– + Lepr^*db*^/+Lepr^*db*^) and *db*/*m* (C57BLKS/J Iar– + m^*db*^/+Lepr^*db*^) mice obtained from the Institute for Animal Reproduction were used for experiments at 8 weeks of age. For the high-fat diet, C57BL/6J mice were fed chow containing 30% fat by weight (14% bovine fat, 14% porcine fat, and 2% soybean oil; Oriental Yeast Co., Tokyo, Japan) from 4 to 24 weeks of age. Mice heterozygous for a floxed *Phd3* allele were generated by injecting *Phd3* floxed embryonic stem cells into C57BL/6N blastocysts. *Phd3*-targeted embryonic stem cell clones were obtained from Helmholtz Zentrum München Deutsches Forschungszentrum für Gesundheit und Umwelt (Augsburg, Germany). To generate liver-specific *Phd3* knockout (KO) mice, we intercrossed mice homozygous for a floxed *Phd3* allele (F/F mice) with α1 anti-trypsin-Cre mice^3^. Male F/F and KO mice were fed normal chow. Primary hepatocytes were prepared from these mice at 8 to 12 weeks of age.

### Cell culture

Primary hepatocytes were isolated from 8- to 12-week-old male C57BL/6 J, F/F, or liver-specific *Phd3* knockout (KO) mice fed normal chow as previously described^17^. Briefly, mice were anaesthetised by intraperitoneal injection of medetomidine (0.75 mg/kg), midazolam (4 mg/kg), and butorphanol (5 mg/kg), and the liver was perfused at a rate of 4.5 ml/min for 3–5 min with oxygenated Hanks’ balanced salt solution containing 10 mM HEPES-NaOH (pH 7.4), followed by 12 min with the same solution containing 30–32 mg/100 ml collagenase type I (CLS-1; Worthington Biochemical, Lakewood, NJ, USA) and Protease Inhibitor Cocktail Complete EDTA-free (one tablet per 50 ml; Roche, Basel, Switzerland). Hepatocytes were harvested and purified by density gradient centrifugation with Percoll (Sigma-Aldrich, St. Louis, MO, USA), and their viability was determined according to Trypan Blue exclusion. Only hepatocyte preparations with a viability >90% were used for experiments. The cells were seeded on type I collagen-coated six-well plates (1.0 × 10^6^/well) in Medium 199 (Life Technologies, Carlsbad, CA, USA) supplemented with 5% foetal bovine serum (FBS) and incubated overnight in serum-free Medium 199 before adding pCPT-cAMP (100 μM) with or without insulin (10 nM), LPS (10 ng/ml), TNF-α (20 ng/ml), DMOG (1 mM), or tunicamycin (5 μg/ml) for the indicated times^63^. Where indicated, primary hepatocytes were exposed to 20 μM H89 for 30 min before incubation in the presence of 100 μM pCPT-cAMP for the indicated times. AML12 and AD-293 cells were obtained from American Type Culture Collection (Manassas, VA, USA) and Agilent Technologies (Santa Clara, CA, USA), respectively. AML12 cells were cultured in a 1:1 (v/v) mixture of Dulbecco’s modified Eagle’s medium (DMEM) and Ham’s F12 medium supplemented with 1× insulin, transferrin, selenium solution (ITS-G, #41400045; Life Technologies); dexamethasone (40 ng/ml); and 10% FBS. AD-293 cells were cultured in DMEM supplemented with 10% FBS. The cell lines were regularly tested for mycoplasma contamination.

### Reagents and antibodies

Glucagon, pCPT-cAMP, insulin, LPS, and MG132 were obtained from Sigma-Aldrich; H-89, DMOG, and tunicamycin were from Enzo Life Sciences (Farmingdale, NY, USA), Merck Calbiochem (Darmstadt, Germany), and Wako Pure Chemical Industries (Osaka, Japan), respectively. Recombinant human TNF-α and murine IL-6 were from Promega (Madison, WI, USA) and Peprotech (Rocky Hill, NJ, USA), respectively. Antibodies used in this study were obtained from Novus Biologicals (Littleton, CO, USA), Cell Signaling Technology (Beverly, MA, USA), Santa Cruz Biotechnology (Dallas, TX, USA), Merck Millipore (Billerica, MA, USA), Sigma-Aldrich, Abcam (Cambridge, UK), BD Biosciences (San Jose, CA, USA), and Transgenic (Fukuoka, Japan), and are listed in Supplementary Table S1.

### Plasmids

PHD3 cDNA was isolated from the liver of a C57/BL6J mouse and cloned into the mammalian expression vector pcDNA3.1 (Life Technologies). The cDNAs for PHD3(ΔPH) (in which Pro^139^ was substituted with Arg to eliminate the prolyl hydroxylase activity) and sh-R PHD3(WT)/(ΔPH) (harbouring an shRNA-resistant three-base substitution within the target sequence) were generated using the KOD-plus Mutagenesis kit (Toyobo, Osaka, Japan). Plasmids expressing HA-CITED2, Myc-GCN5, and FLAG-GCN5 have been previously described^8^.

### Adenoviruses

Recombinant adenoviruses were constructed using an Adenovirus dual expression kit (Takara Bio, Otsu, Japan). FLAG-PHD3(WT), sh-R FLAG-PHD3(WT), sh-R FLAG-PHD3(ΔPH), Cre, and LacZ (control) were expressed under the control of a CAG promoter, whereas shRNAs were expressed under the control of the U6 promoter. The shRNA for PHD3 was based on the sequence 5′-GGAAATCGTTTGTAGCAGA-3′. A negative control shRNA sequence was obtained from BD Biosciences. Adenoviral vectors encoding CITED2, GCN5, or PGC-1α shRNA and FLAG-CITED2, FLAG-GCN5, HA-CITED2, and FLAG-PGC-1α have been previously described^8,22^. Primary hepatocytes or AML12 cells were infected with adenoviruses 1 day after plating. Analyses of gene and protein expression and protein-protein interaction and the glucose production assay were carried out 2 days after infection.

### RNA preparation and quantitative real-time (qRT)-PCR

Total RNA was isolated from cells or pulverised tissue using NucleoSpin RNA filters (Macherey-Nagel, Düren, Germany). For qRT-PCR analysis, cDNA was synthesised from total RNA using random primers and a High Capacity cDNA Reverse Transcription kit (Thermo Fisher Scientific, Waltham, MA, USA), and PCR was performed in triplicate on a StepOnePlus Real-Time PCR System using Fast SYBR Green Master Mix (Thermo Fisher Scientific). Relative mRNA abundance was calculated by the standard curve method and was normalised to the corresponding amount of 18S rRNA. Primer sequences are listed in Supplementary Table S2. Unlisted primer sequences have been previously described^8^.

### Preparation of total protein extract from cells and tissue

Epitope-tagged or untagged proteins and/or shRNAs were expressed in mouse hepatocytes, AML12 cells, or AD-293 cells by transfection using X-tremeGENE HP (Roche) or by adenoviral transduction. The cells were then lysed in lysis buffer composed of 20 mM Tris-HCl (pH 7.5), 150 mM NaCl, 0.5% Nonidet P (NP)-40, 2 mM EDTA, 10% glycerol, and protease and phosphatase inhibitor cocktails (Roche). The lysates were centrifuged at 15,000 × *g* for 20 min at 4 °C and the supernatant was collected. Proteins were denatured by boiling at 98 °C for 5 min in 1 × Laemmli buffer. Liver, adipose, and muscle tissues (50–100 mg) were homogenised with TissueLyser II (Qiagen, Valencia, CA, USA) in 1 ml ice-cold lysis buffer A composed of 20 mM Tris-HCl (pH 7.5), 1% NP-40, 10% glycerol, 137 mM NaCl, 1 mM MgCl_2_, 2.5 mM CaCl_2_, 1 mM dithiothreitol, 1 mM phenylmethylsulfonyl fluoride, 1 mM Na_3_VO4, 1 mM EDTA, 50 mM NaF, 10 mM NaPPi, and protease and phosphatase inhibitor cocktails (Roche). After homogenisation, lysates were centrifuged at 15,000 × *g* for 20 min. After carefully removing the top lipid layer, the protein concentration of the lysates was determined using a Pierce bicinchoninic acid protein assay kit (Thermo Fisher Scientific) and equalised across samples by adding the appropriate volume of lysis buffer. Proteins were denatured in 1 × Laemmli buffer by boiling at 98 °C for 5 min. Nuclear extracts were prepared using the CelLytic NuCLEAR Extraction kit (Sigma-Aldrich) according to the manufacturer’s protocol using IGEPAL CA-630.

### Immunoprecipitation

For immunoprecipitation, cells were lysed in lysis buffer A. Cell lysates were cleared by centrifugation and subjected to immunoprecipitation with the indicated antibodies (1–8 μg/sample) and Dynabeads Protein G (1.5 mg/sample, Thermo Fisher Scientific) at 4 °C for 3 h. Beads were precipitated with a magnet and washed three times with 1 ml ice-cold wash buffer composed of 20 mM Tris-HCl (pH 7.5), 1% NP-40, 150 mM NaCl, and 2 mM EGTA. The pellet was resuspended in 2 × Laemmli buffer and incubated at 98 °C for 5 min.

### Immunoblotting

Cell or tissue lysates and immunoprecipitates prepared as described above were resolved by sodium dodecyl sulphate polyacrylamide gel electrophoresis. The proteins were then transferred to a nitrocellulose membrane that was blocked in Tris-buffered saline with 0.05% Tween 20 (TBST; pH 7.4) and 5% skim milk for 1 h at room temperature, followed by incubation with primary antibody in TBST with 10% foetal calf serum and 1% bovine serum albumin at 4 °C for 3 h or overnight. The membrane was washed three times in TBST and incubated with secondary antibody in TBST with 0.5% skim milk for 1 h at room temperature. After three washes in TBST, the membrane was developed using SuperSignal West Pico Chemiluminescent Substrate (Thermo Fisher Scientific) and visualised on a LAS4000mini system (Fujifilm, Tokyo, Japan). The membrane was stripped by vigorous shaking in Western Blot Stripping Buffer (Takara Bio) at room temperature for 15–30 min, followed by three 10-min washes in TBST.

### Statistical analysis

Quantitative data are presented as mean ± SEM and were analysed using Prism software (GraphPad, La Jolla, CA, USA). Each experiment was performed at least three times. Results were evaluated with the two-tailed Student’s *t*-test or by one- or two-way analysis of variance (ANOVA) as appropriate. Welch’s *t* test was used when the variance differed among groups. Significant differences detected by ANOVA were assessed with the Bonferroni post hoc test. P < 0.05 was considered statistically significant.

## Electronic supplementary material

Supplementary information

## Data Availability

Data supporting the findings of this study are available by request from the corresponding author.
