# Supplementary material for: PHD3 regulates glucose metabolism by suppressing stress-induced signalling and optimising gluconeogenesis and insulin signalling in hepatocytes
Source: Sci Rep. 2018 Sep 24;8:14290. doi: 10.1038/s41598-018-32575-z (PMC6155226; doi:10.1038/s41598-018-32575-z)

Supplementary Information

PHD3 regulates glucose metabolism by suppressing stress-induced signalling and optimising gluconeogenesis and insulin signalling in hepatocytes

Hiroyuki Yano[1,2], Mashito Sakai[1], Toshiya Matsukawa[1], Takashi Yagi[1,2], Takao Naganuma[1], Masaru Mitsushima[1], Satoshi Iida[1], Yuka Inaba[3], Hiroshi Inoue[3], Hiroyuki Unoki-Kubota[4], Yasushi Kaburagi[4], Shun-ichiro Asahara[5], Yoshiaki Kido[5,6], Shiro Minami[2], Masato Kasuga[7], Michihiro Matsumoto[1]

[1]Department of Molecular Metabolic Regulation, Diabetes Research Center, Research Institute, National Center for Global Health and Medicine, Tokyo 162-8655, Japan

[2]Department of Bioregulation, Institute for Advanced Medical Sciences, Nippon Medical School, Kawasaki 211-8533, Japan

[3]Metabolism and Nutrition Research Unit, Innovative Integrated Bio-research Core, Institute for Frontier Science Initiative, Kanazawa University, Kanazawa 920-8641, Japan

[4]Department of Diabetic Complications, Diabetes Research Center, Research Institute, National Center for Global Health and Medicine, Tokyo 162-8655, Japan

[5]Division of Diabetes and Endocrinology, Department of Internal Medicine, Kobe University Graduate School of Medicine, Kobe 650-0017, Japan

[6]Division of Metabolism and Disease, Department of Biophysics, Kobe University Graduate School of Health Sciences, Kobe 654-0142, Japan

[7]National Center for Global Health and Medicine, Tokyo 162-8655, Japan

Supplementary Methods

Glucose production assay

The assay was carried out as previously described[1]. Briefly, primary hepatocytes were cultured in serum-free Medium 199 in the absence or presence of 100 μM pCPT-cAMP for 16 h, then incubated for 6 h in glucose- and Phenol Red-free DMEM (pH 7.4) supplemented with sodium lactate and pyruvate before measurement of glucose released into the medium with a colorimetric assay. Data are presented as arbitrary units.

Supplementary figure legends

Supplementary Figure S1. Related to Figure 1. (**A**) qRT-PCR detection of mRNA levels of three *Phd* isoforms and *Vegf* in primary hepatocytes with or without exposure to glucagon (100 nM, 6 h) or pCPT-cAMP (100 μM, 6 h). (**B**) qRT-PCR analysis of *Phd3* mRNA in primary hepatocytes with or without exposure to pCPT-cAMP (100 μM, 6 h) or in the presence or absence of insulin (10 nM, 6 h). Data are shown as mean ± SEM (n = 3) and are representative of at least two independent experiments. Data were evaluated by ANOVA followed by Bonferroni's post hoc test. **P < 0.01 vs. indicated groups.

Supplementary Figure S2. Related to Figure 2. (**A**) *Cited2* mRNA level in primary hepatocytes with or without CITED2 knockdown and exposure to pCPT-cAMP (100 μM, 6 h), as detected by qRT-PCR. See Figure 2A. (**B**) qRT-PCR analysis of *Cited2* mRNA level in primary hepatocytes with or without CITED2 overexpression and with or without exposure to pCPT-cAMP (100 μM, 6 h). See Figure 2B. (**C**) qRT-PCR analysis of *Gcn5* mRNA level in primary hepatocytes with or without GCN5 knockdown and exposure to pCPT-cAMP (100 μM, 6 h). See Figure 2C. (**D**) qRT-PCR analysis of *Ppargc1a* mRNA level in primary hepatocytes with or without PGC-1α knockdown and exposure to pCPT-cAMP (100 μM, 6 h).

See Figure 2D. Data are shown as mean ± SEM (n = 3) and are representative of at least two independent experiments. Differences were evaluated by ANOVA followed by Bonferroni's post hoc test. **P < 0.01 vs. indicated groups.

Supplementary Figure S3. Related to Figure 3. (**A**) PHD3 mRNA level in primary hepatocytes with or without PHD3 knockdown and exposure to pCPT-cAMP (100 μM, 6 h), as detected by qRT-PCR. See Figure 3A. (**B**) Gluconeogenic enzyme gene and PHD3 mRNA expression in primary hepatocytes isolated from control PHD3 flox/flox mice (F/F) or liver-specific PHD3 knockout (PHD3 flox/flox; α1-anti-trypsin-Cre) mice (KO) with or without exposure to pCPT-cAMP (100 μM, 6 h), as detected by qRT-PCR. (**C**) Gluconeogenic enzyme gene and PHD3 mRNA levels in hepatocytes isolated from PHD3 flox/flox mice transduced with adenoviral vector encoding Cre recombinase or control β-galactosidase, with or without exposure to pCPT-cAMP (100 μM, 6 h). (**D**) Immunoblot analysis of PHD3 in primary mouse hepatocytes with ectopic expression of either shRNA-resistant PHD3(WT) or PHD3(ΔPH) with or without shRNA-mediated PHD3 knockdown in the presence of pCPT-cAMP (100 μM, 6 h). See Figure 3C. (**E**) Immunoblot analysis of HIF-2α in primary hepatocytes isolated from F/F, KO, or C57BL/6N (WT) mice with or without exposure to DMOG (1 mM, 4h). α-Tubulin served as the loading control for immunoblotting. Complete immunoblots is presented in Supplementary Figure S7. Quantitative data are shown as mean ± SEM (n = 3) and are representative of at least two independent experiments. Differences between groups were evaluated with the unpaired Student's t test (*Phd3* in B and C) or by ANOVA followed by Bonferroni's post hoc test (A, and *G6pc* and *Pck1* in B and C). **P < 0.01 vs. control or as indicated.

Supplementary Figure S4. Related to Figure 4. (**A**) qRT-PCR analysis of *Ppargc1a* (PGC-1α) mRNA level in primary mouse hepatocytes expressing FLAG-PGC-1α (or not) with or without PHD3 shRNA. See Figure 4F.

Supplementary Figure S5. Related to Figure 5. (**A**) Primary hepatocytes isolated from control PHD3 flox/flox mice (F/F) or liver-specific PHD3 knockout mice (KO) were exposed to 10 nM insulin for 1 min or left untreated, and then subjected to immunoblot analysis with antibodies to Tyr^{1146}-phosphorylated IRβ or native IRβ. The cells were also subjected to immunoprecipitation with antibodies against IRS-1 or -2, followed by immunoblotting with antibodies against phosphorylated tyrosine (αPY), p85 subunit of PI3K, or IRS-1 or -2. *IgG heavy chain. (**B**) Primary hepatocytes isolated from control PHD3 flox/flox mice (F/F) or liver-specific PHD3 knockout mice (KO) were exposed to 10 nM insulin for 10 min or left untreated and then subjected to immunoblot analysis with antibodies against Thr^{308}- or Ser^{473}-phosphorylated Akt, Akt, $Ser^{21/9}$-phosphorylated GSK-3α/β, GSK-3β, or α-tubulin (loading control for immunoblotting). (**C**) Effects of ectopic expression of WT or mutant forms of PHD3 on insulin-induced (10 nM, 1 min) Tyr^{1146} phosphorylation and IRβ protein level, and insulin-induced (10 nM, 10 min) phosphorylation of Akt at Thr^{308} and Ser^{473} and GSK-3α/β at $Ser^{21/9}$, and Akt and GSK-3β protein levels in primary mouse hepatocytes, as determined by immunoblotting. (**D**) qRT-PCR detection of *Phd3* mRNA in primary mouse hepatocytes with or without shRNA-mediated PHD3 knockdown used in Figure 5F and G and Supplementary Figure S5E. (**E**) Effects of PHD3 depletion on insulin-induced (10 nM, 10 min) phosphorylation of p70 S6 kinase at Thr^{389} and Thr^{421}/Ser^{424} and p70 S6 kinase level in primary mouse hepatocytes, as determined by immunoblotting. Complete immunoblots are presented in Supplementary Figure S7. Quantitative data are shown as mean ± SEM (n = 3)

and are representative of at least two independent experiments. Differences between groups were evaluated with the unpaired Student’s t test. **P < 0.01 vs. control.

Supplementary Figure S6. Related to Figure 6. (**A**) mRNA expression of proinflammatory genes in primary hepatocytes isolated from control PHD3 flox/flox (F/F) or liver-specific PHD3 knockout (PHD3 flox/flox; α1-anti-trypsin-Cre) (KO) mice with or without exposure to 100 ng/ml LPS for 2 h, as detected by qRT-PCR. (**B**) qRT-PCR detection of proinflammatory gene and *Phd3* transcript levels in hepatocytes isolated from PHD3 flox/flox mice transduced with adenoviral vector encoding Cre recombinase or control β-galactosidase. (**C**) qRT-PCR detection of *Phd3* mRNA level in primary mouse hepatocytes with ectopic expression of shRNA-resistant PHD3(WT) or PHD3(ΔPH) with shRNA-mediated PHD3 knockdown (see Fig. 6C). Quantitative data are shown as mean ± SEM (n = 3) and are representative of at least two independent experiments. Differences between groups were evaluated with the unpaired Student’s t test (B) or by ANOVA followed by Bonferroni’s post hoc test (A, C). *P < 0.05, **P < 0.01 vs. control or as indicated.

Supplementary References

1. Matsumoto, M. & Sakai, M. Glucose production assay in primary mouse hepatocytes. Bio-protocol **2**, e284 (2012).

Supplementary Table S1

Antibodies used for immunoprecipitation (IP) and western blotting (WB)

Antibodies	Dilution	Cat#, Company
Anti-PHD3	1:1000 WB	NB100-303, Novus Biologicals
Anti-PHD3	1:1000 WB	NB100-139, Novus Biologicals
Anti-phospho-IP3 receptor (Ser1756)	1:1000 WB	#8548, Cell Signaling Technology (CST)
Anti-IP3 receptor 1	1:1000 WB	#8568, CST
Anti-phospho-CREB (Ser133)	1:1000 WB	#9198, CST
Anti-CREB	1:1000 WB	#9197, CST
Anti-phospho-VASP (Ser157)	1:1000 WB	#3111, CST
Anti-VASP	1:1000 WB	#3132, CST
Anti-Myc-tag	1:1000 WB	#2272, CST
Anti-phospho-(Ser/Thr) PKA substrate	1:50 IP; 1:1000 WB	#9621, CST
Anti-phospho-Akt (Thr308)	1:1000 WB	#9275, CST
Anti-phospho-Akt (Ser473)	1:1000 WB	#9271, CST
Anti-Akt (pan)	1:1000 WB	#4691, CST
Anti-phospho-GSK3β (Ser21/9)	1:1000 WB	#9331, CST
Anti-phospho-p44/42 MAPK (Erk1/2) (Thr202/204)	1:1000 WB	#9101, CST
Anti-p44/42 MAPK (Erk1/2)	1:1000 WB	#9102, CST
Anti-phospho-insulin receptor β (Tyr1146)	1:1000 WB	#3021, CST
Anti-phospho-NF-κB p65 (Ser536)	1:1000 WB	#3033, CST
Anti-phospho-SAPK/JNK (Thr183/Tyr185)	1:1000 WB	#9255, CST
Anti-JNK2	1:1000 WB	#9258, CST
Anti-phospho-Stat3 (Tyr705)	1:1000 WB	#9138, CST
Anti-phospho-p70 S6 kinase (Thr421/Ser424)	1:1000 WB	#9204, CST
Anti-phospho-p70 S6 kinase (Thr389)	1:1000 WB	#9205, CST
Anti-p70 S6 kinase	1:1000 WB	#9202, CST
Anti-phospho-eIF2α (Ser51)	1:1000 WB	#9721, CST
Anti-eIF2α	1:1000 WB	#9722, CST
Anti-phospho-PERK (Thr980)	1:1000 WB	#3179, CST
Anti-PERK	1:1000 WB	#3192, CST
Anti-histone H3	1:1000 WB	#4499, CST
Anti-acetylated lysine	1:1000 WB	#9441, CST
Anti-GCN5	1:500 WB	sc-20698, Santa Cruz Biotechnology
Anti-insulin receptor β	1:500 WB	sc-711, Santa Cruz Biotechnology
Anti-NF-κB p65	1:1000 WB	sc-372, Santa Cruz Biotechnology
Anti-Stat3	1:1000 WB	sc-482, Santa Cruz Biotechnology
Anti-XBP-1	1:200 WB	sc-7160, Santa Cruz Biotechnology
Anti-CREB-2 (for ATF-4)	1:200 WB	sc-200, Santa Cruz Biotechnology
Anti-PGC-1α	1:500 WB	sc-13067, Santa Cruz Biotechnology
Anti-phosphotyrosine (clone 4G10)	1:2000 WB	05-321, Merck Millipore
Anti-IRS-1	1:60 IP; 1:1000 WB	06-248, Merck Millipore
Anti-IRS-2	1:60 IP; 1:1000 WB	06-506, Merck Millipore
Anti-PI3 kinase, p85	1:1000 WB	06-497, Merck Millipore
Anti-α-tubulin	1:8000 WB	T6199, Sigma-Aldrich
Anti-HA	1:2000 WB	11867423001, Sigma-Aldrich
Anti-α-tubulin	1:10000 WB	ab7291, Abcam
Anti-HIF-1α	1:500 WB	ab2185, Abcam
Anti-HIF-2α	1:500 WB	ab199, Abcam
Anti-GSK-3β	1:2000 WB	610202, BD Biosciences
Anti-DYKDDDDK (for FLAG)	1:50 IP; 1:2000 WB	KO602, Transgenic

Primers used for qRT-PCR

Gene	Forward	Reverse
Phd1	5'-ATTGCCTGGGTAGAAGGTCACG-3'	5'-GAGCCCATTGCCTGGATAACAC-3'
Phd2	5'-CTACAGGATAAACGGCCGAACG-3'	5'-CACTTACCTTGGCGTCCCAGTC-3'
Phd3	5'-CTTCCTCCTGTCCCTCATCGAC-3'	5'-TCCTGGATAGCAAGCCACCATT-3'
Vegf	5'-TTCTGCTCTCTTGGGTGCACTG-3'	5'-CTCAATCGGACGGCAGTAGCTT-3'
Nr4a1	5'-TGACAATGCTTCGTGTCAGCA-3'	5'-TCCACAGGGCAATCCTTGTTT-3'
Nr4a2	5'-CATCAGAGGGTGGGCAGAGAA-3'	5'-CAAGACCACCCCATTGCAAAA-3'
Nr4a3	5'-ACGCCGAAACCGATGTCAGTA-3'	5'-TCGGACAAGGGCATTCATCAT-3'
Sirt1	5'-CAGTGTCATGGTTCCTTTGC-3'	5'-CACCGAGGAACTACCTGAT-3'
Gpt	5'-TGCAGGGCGCTATGTATTCCTT-3'	5'-CACAACGCAGATGCCAGTCTCT-3'
Srebf1c	5'-GAAGCTGTCGGGGTAGCGTCT-3'	5'-CTCTCAGGAGAGTTGGCACCTG-3'
Dgat1	5'-CTAGTGAGCGTTCCCCTGCG-3'	5'-ACCGGTTGCCCAATGATGAG-3'
Irs1	5'-CCAGCTCCACCCAGCTCCTA-3'	5'-TGCCCAACTCAACTCCACCA-3'
Irs2	5'-CAGGAGCAGGAGGGCTGGTA-3'	5'-GGAGAGAGGCGCTGCAAGAG-3'
Tnf	5'-CTATGGCCCAGACCCTCACACT-3'	5'-CACTCCAGCTGCTCCTCCACTT-3'
Il6	5'-TGGAGCCCACCAAGAACGATAG-3'	5'-ACCAGCATCAGTCCCAAGAAGG-3'
Nos2	5'-CCACACACTGGCCTCCCTCT-3'	5'-ACGGGCCCGGTACTCATTCT-3'
Socs1	5'-GACACTCACTTCCGCACCTTCC-3'	5'-GCTCAGGGGTCCCCAATAGAAG-3'
Socs3	5'-AGCTGCAGGAGAGCGGATTCTA-3'	5'-GCTGTCGCGGATAAGAAAGGTG-3'
Atf4	5'-GTCTCCTCGGCCCAAACCTTAT-3'	5'-TGGCTGCTGTCTTGTTTTGCTC-3'
Ddit3	5'-CCTCGCTCTCCAGATTCCAGTC-3'	5'-TGGGCACTGACCACTCTGTTTC-3'

Supplementary Figure S1 (Related to Figure 1)

A

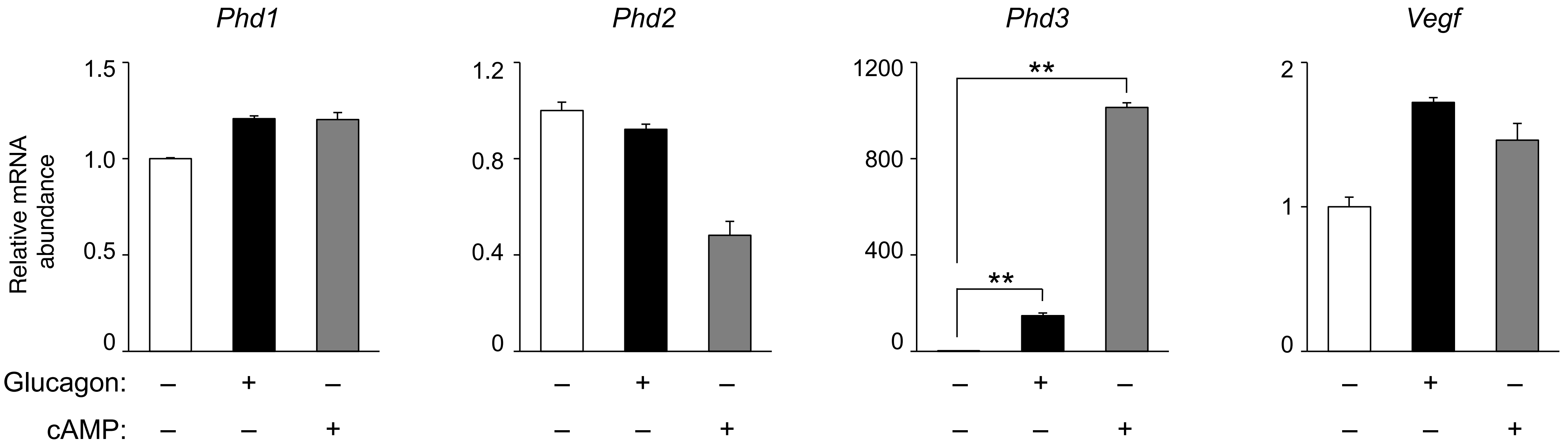

B

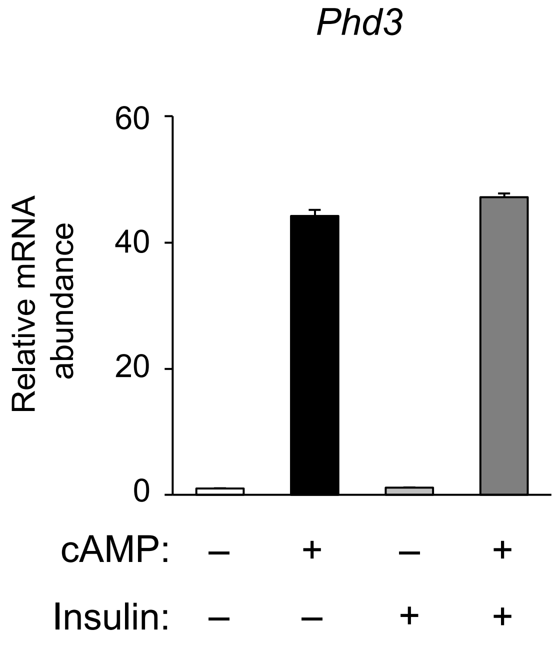

Supplementary Figure S2 (Related to Figure 2)

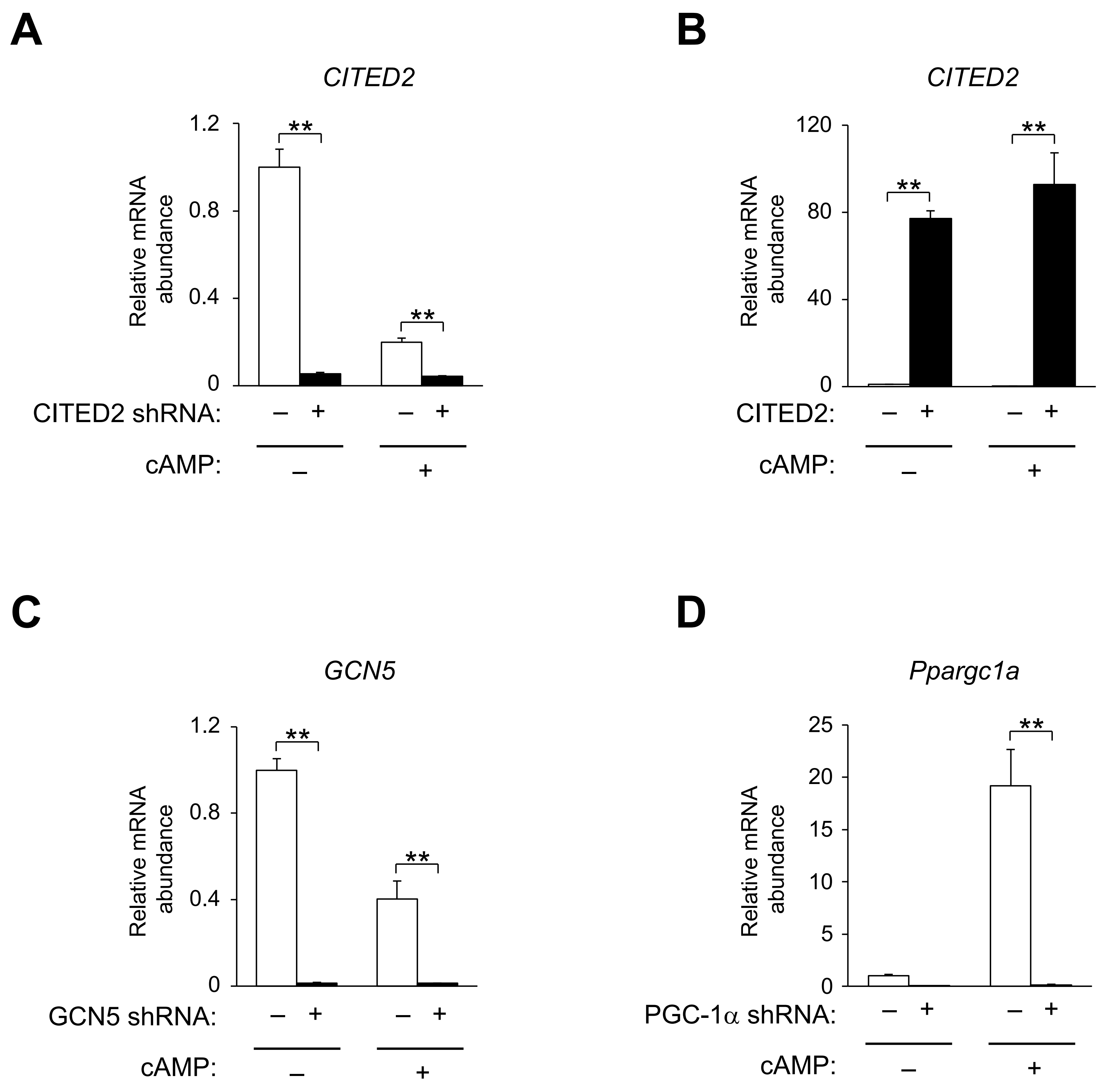

Supplementary Figure S3 (Related to Figure 3)

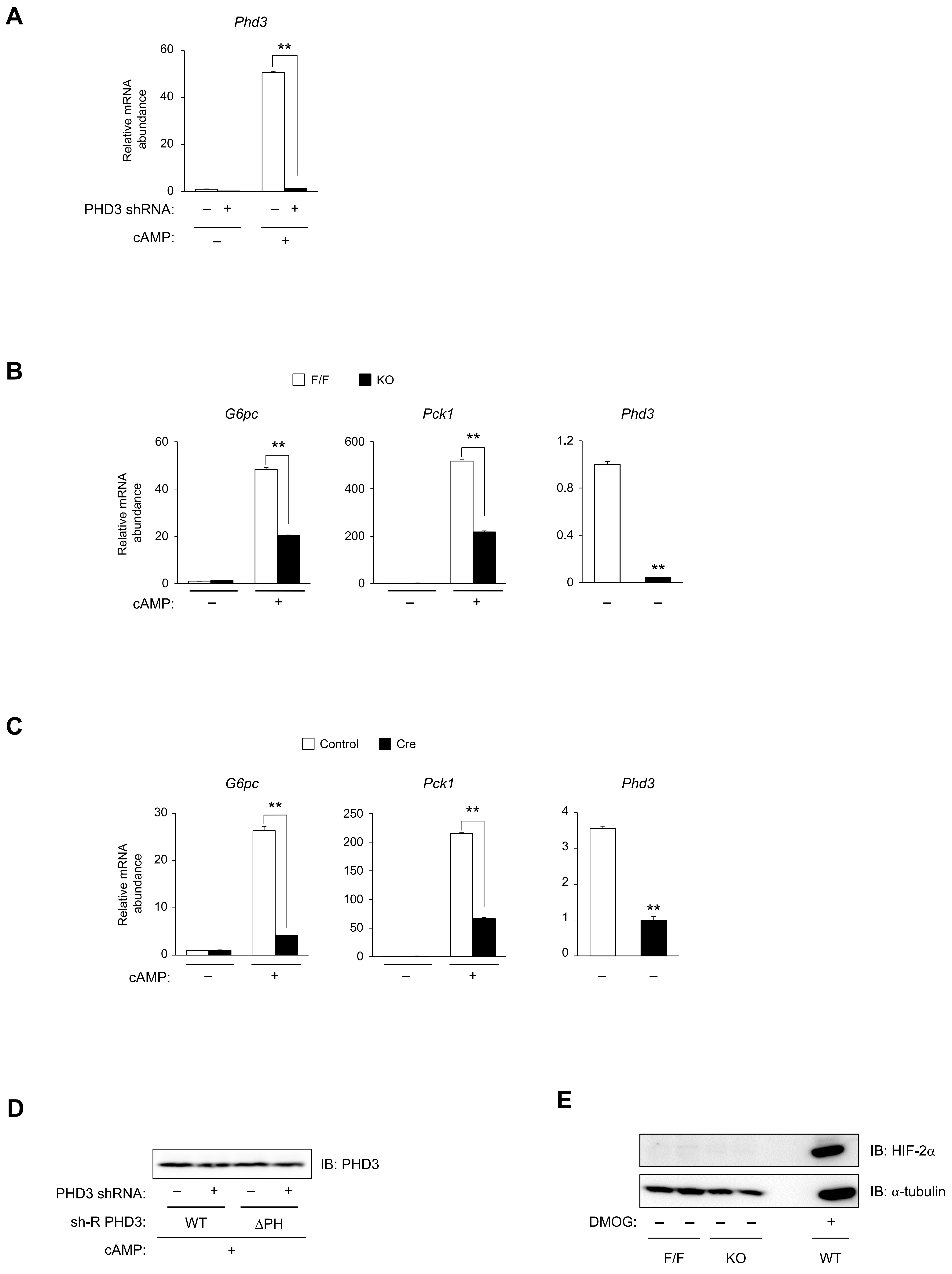

Supplementary Figure S4 (Related to Figure 4)

A

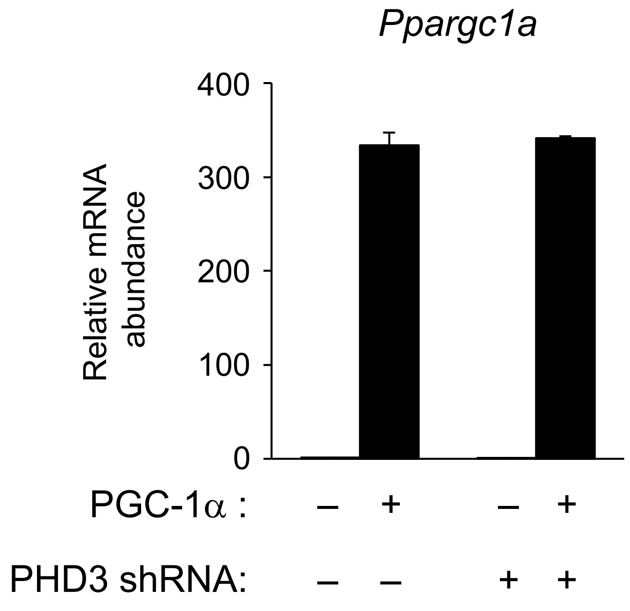

Supplementary Figure S5 (Related to Figure 5)

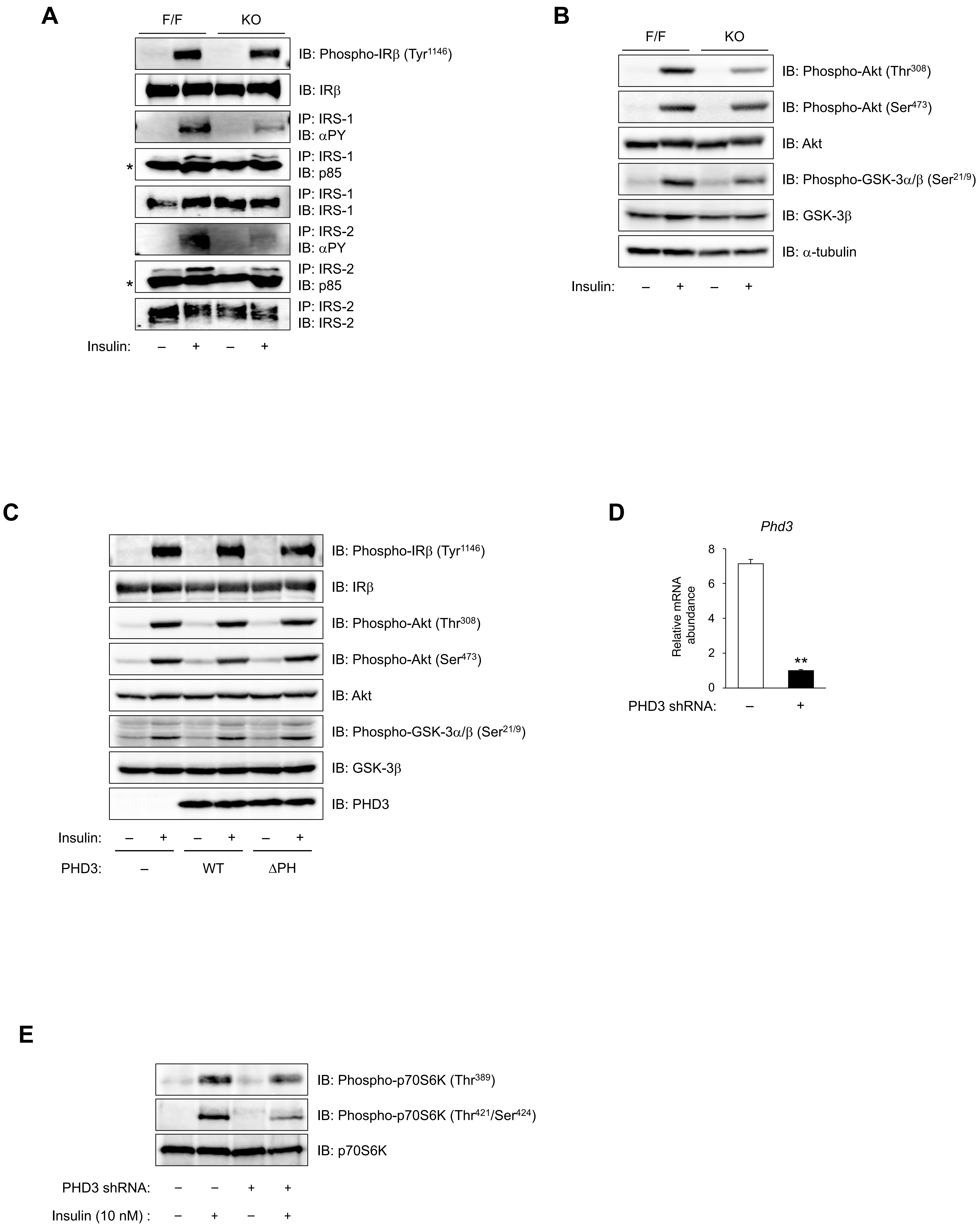

Supplementary Figure S6 (Related to Figure 6)

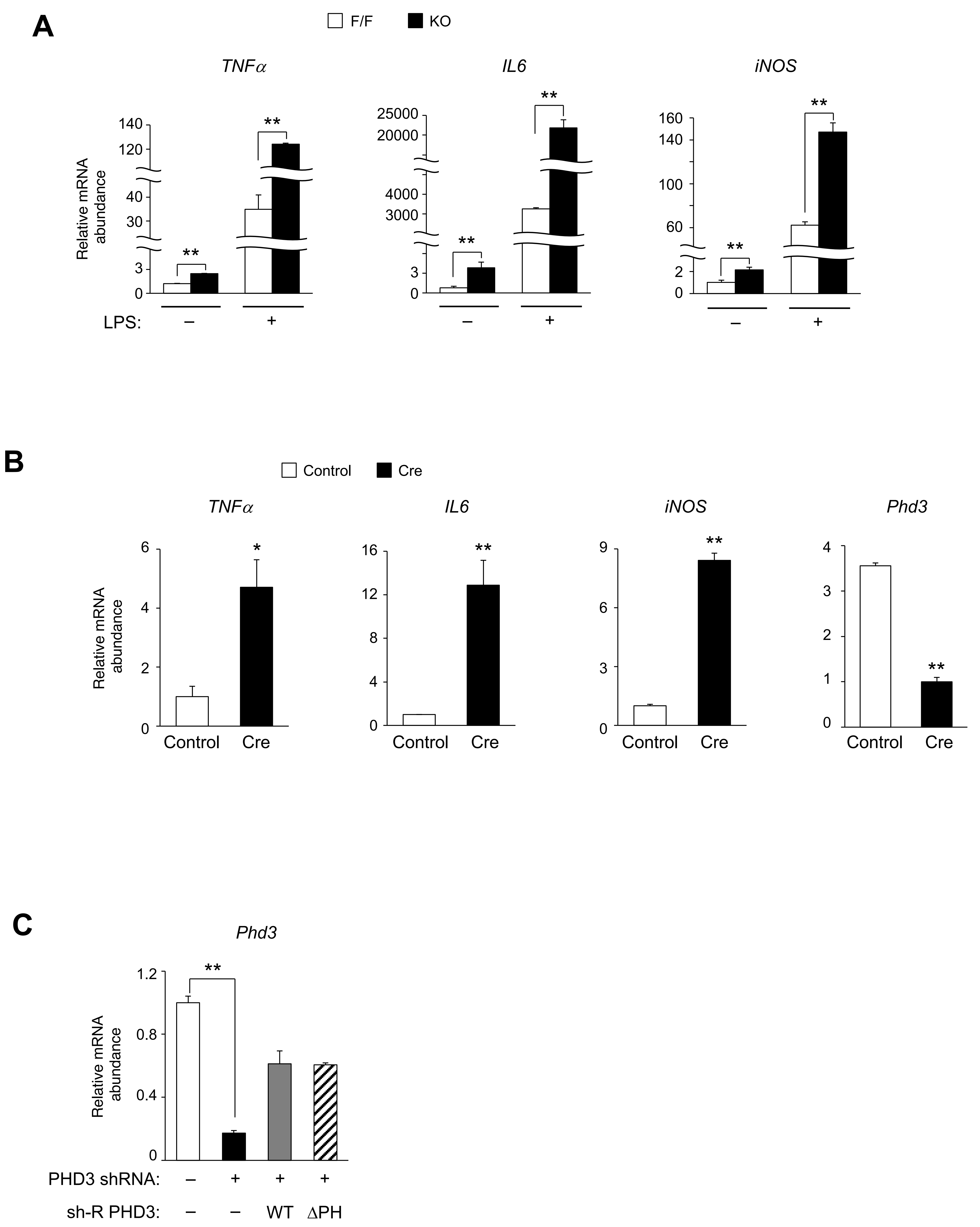

Supplementary Figure S7

Full-length images of original scans of representative immunoblots

Figure 1C

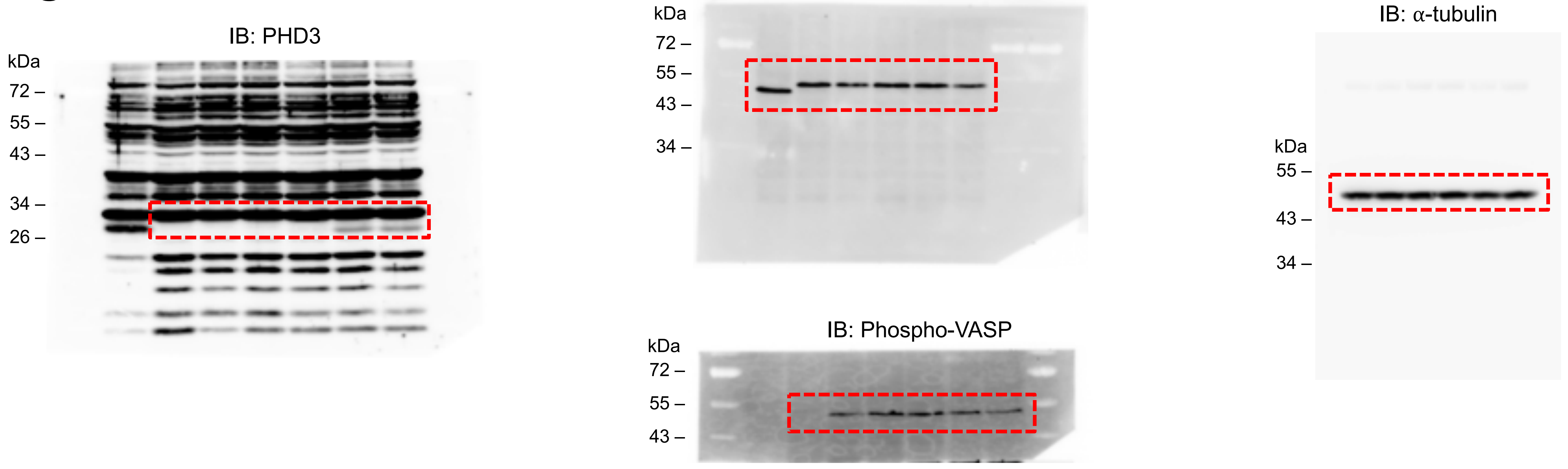

Figure 1G

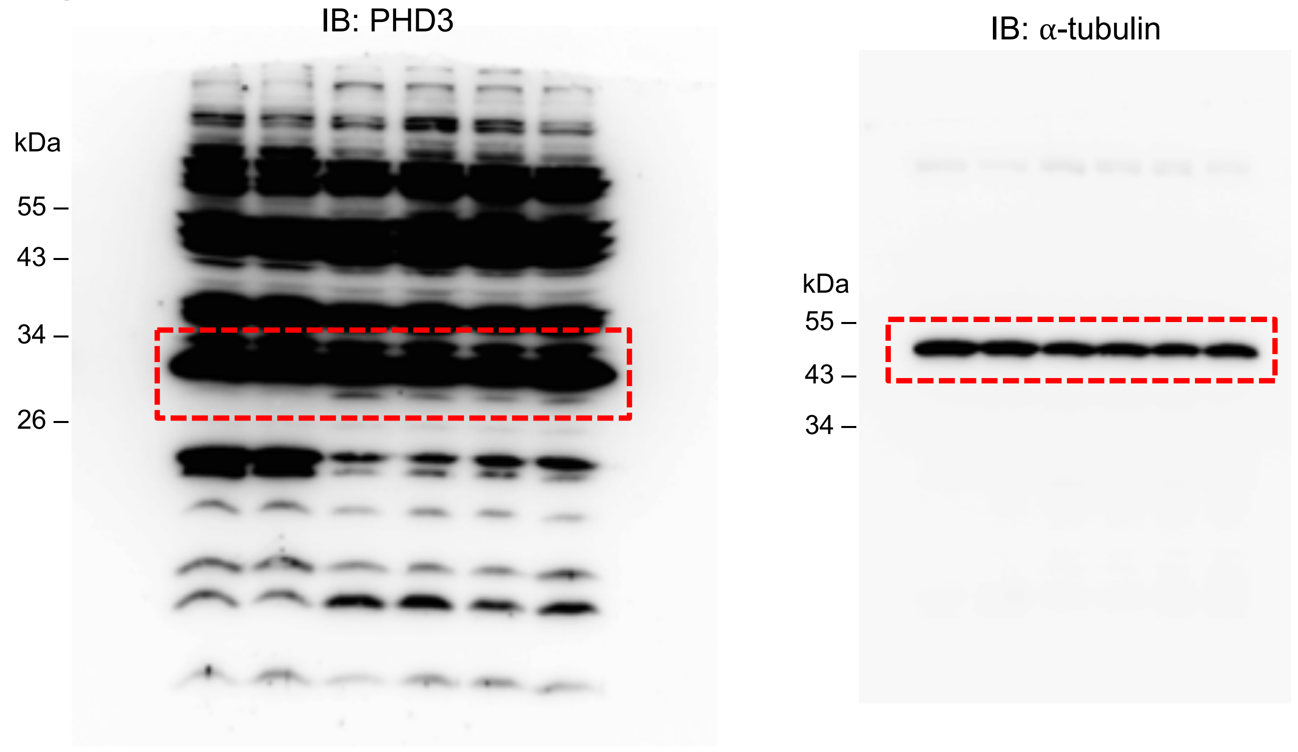

Figure 3E

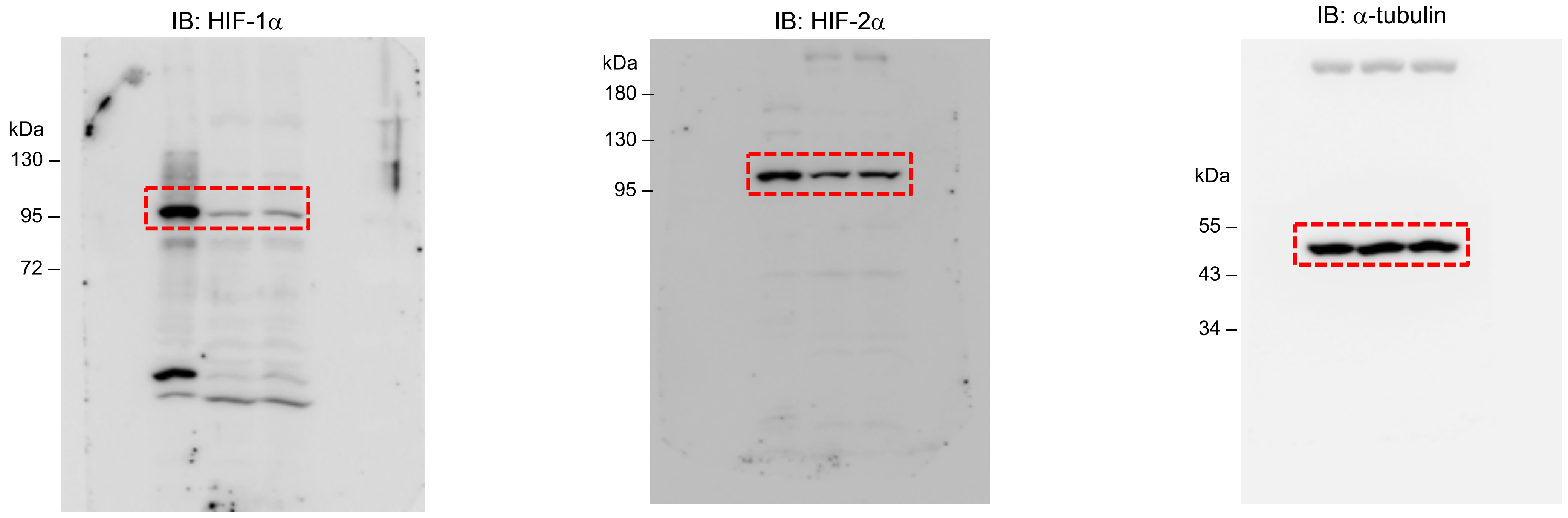

Figure 4A (Continued on next page)

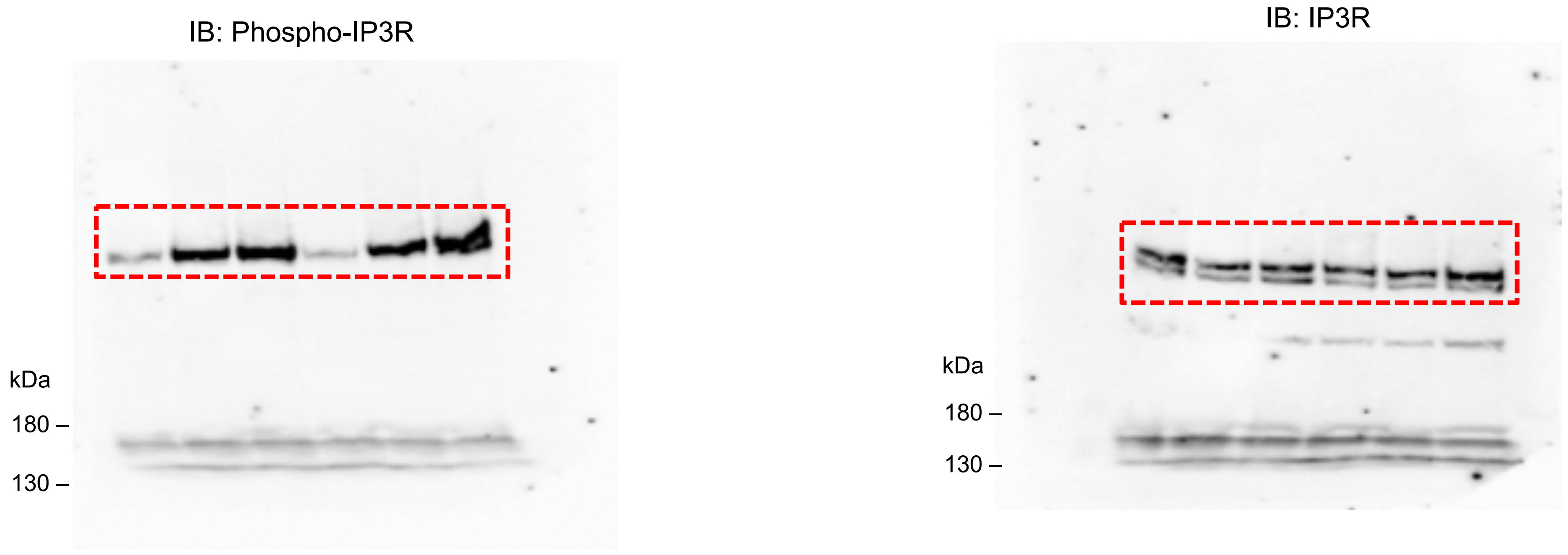

Supplementary Figure S7 (Continued on next page)

Figure 4A

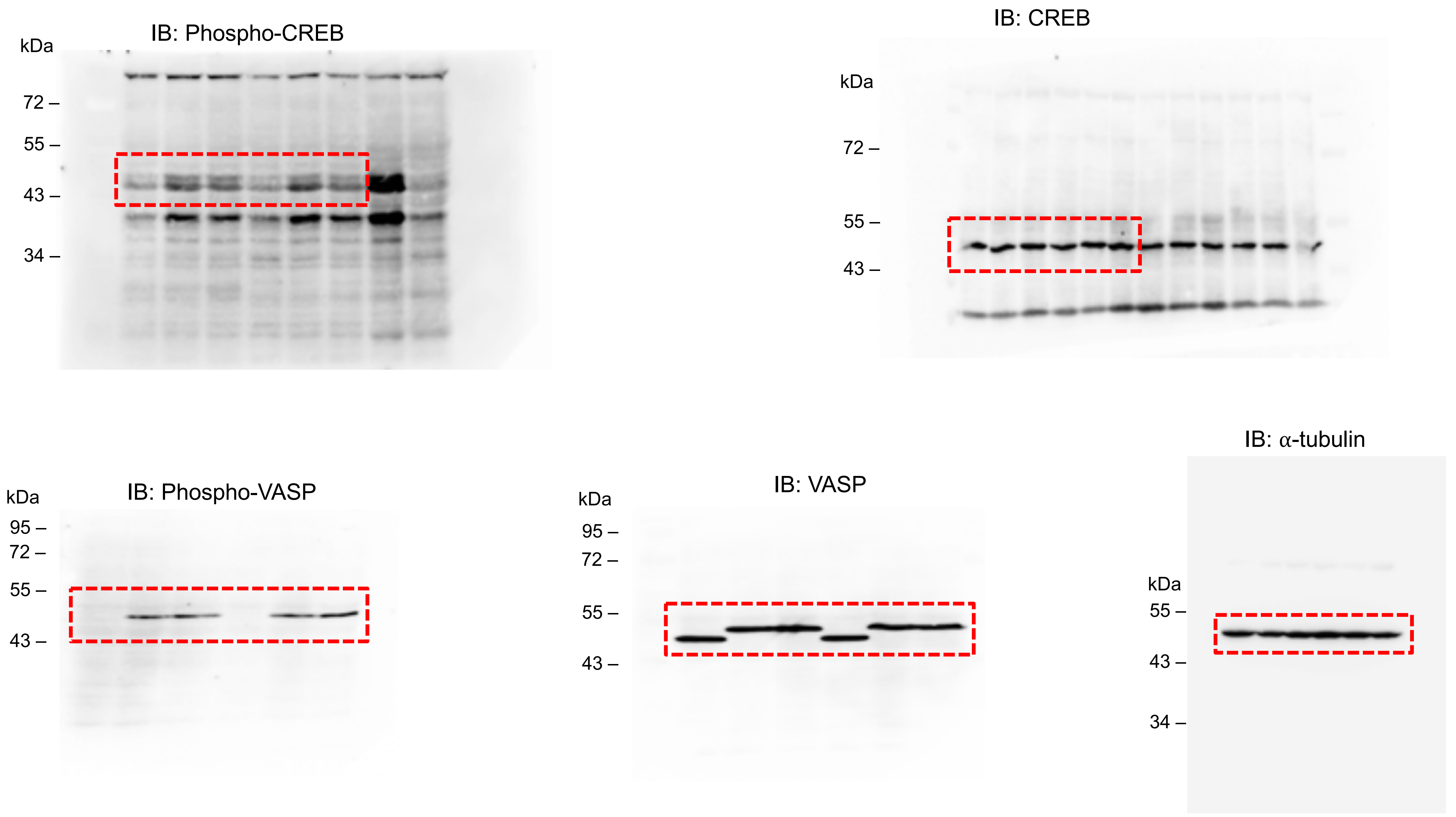

Figure 4B

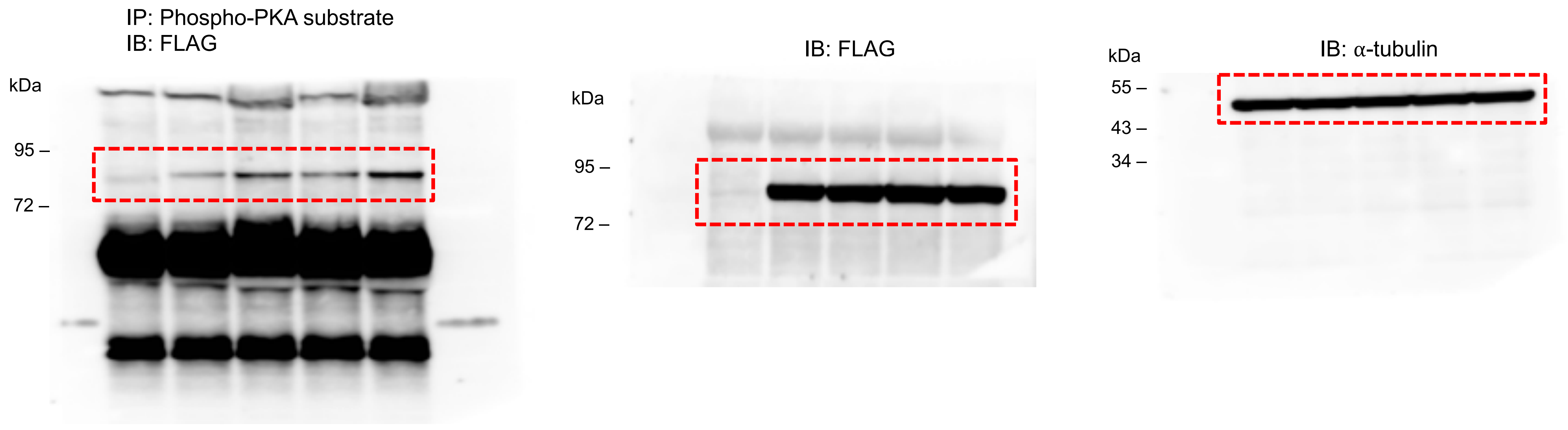

Figure 4C

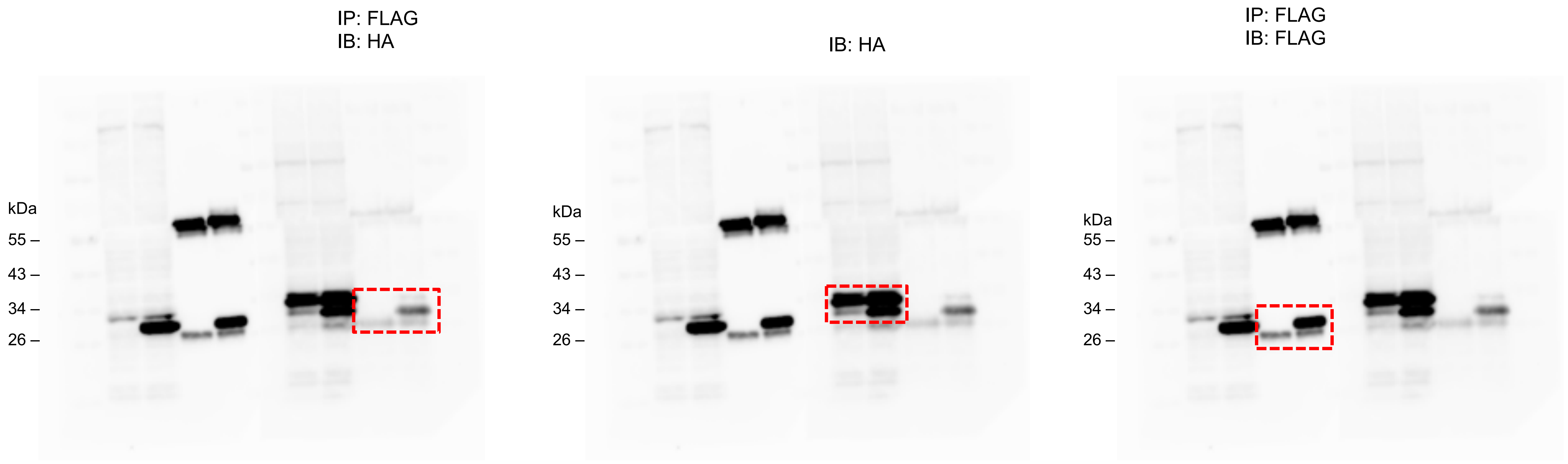

Supplementary Figure S7 (Continued on next page)

Figure 4D

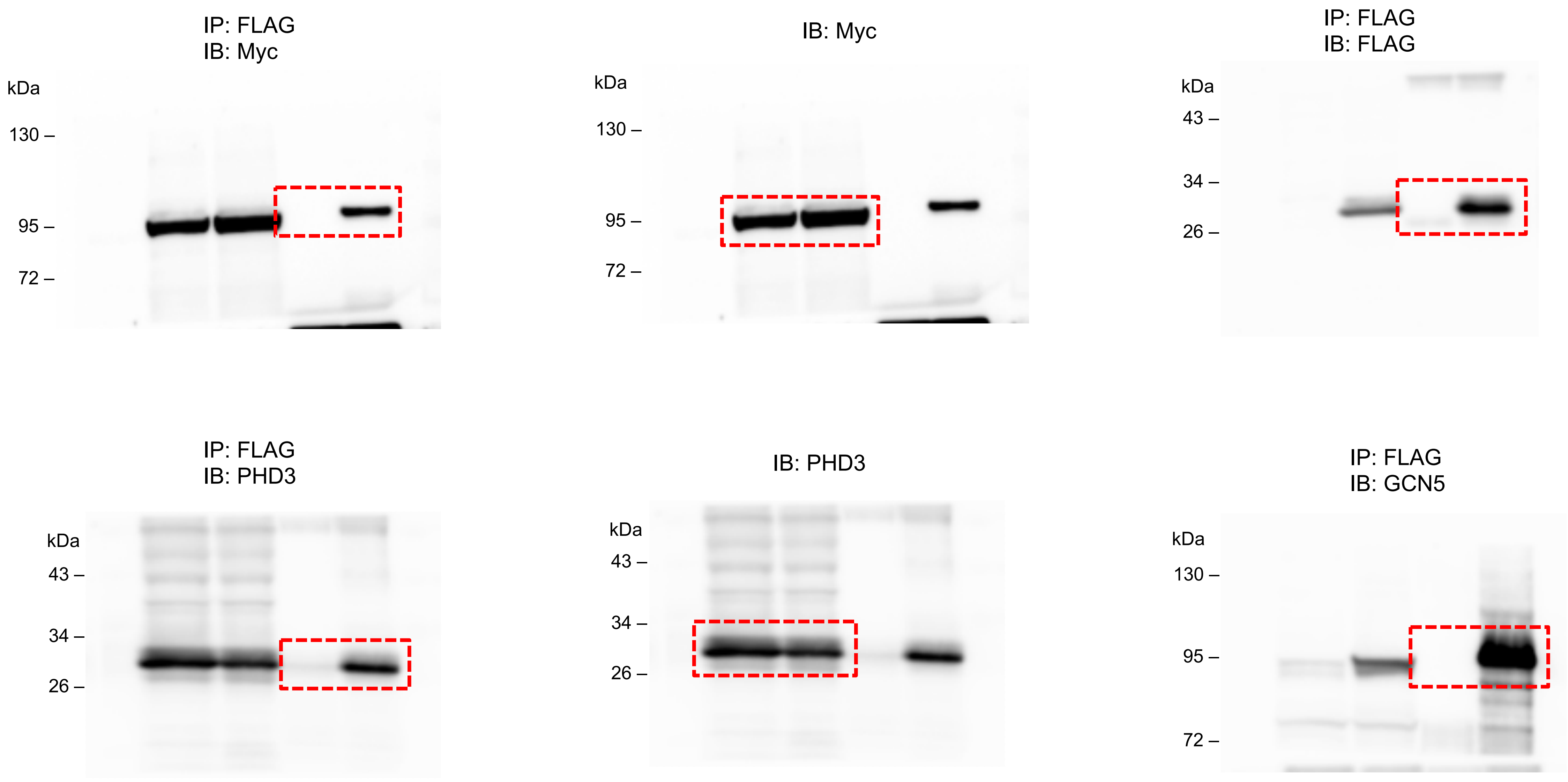

Figure 4E

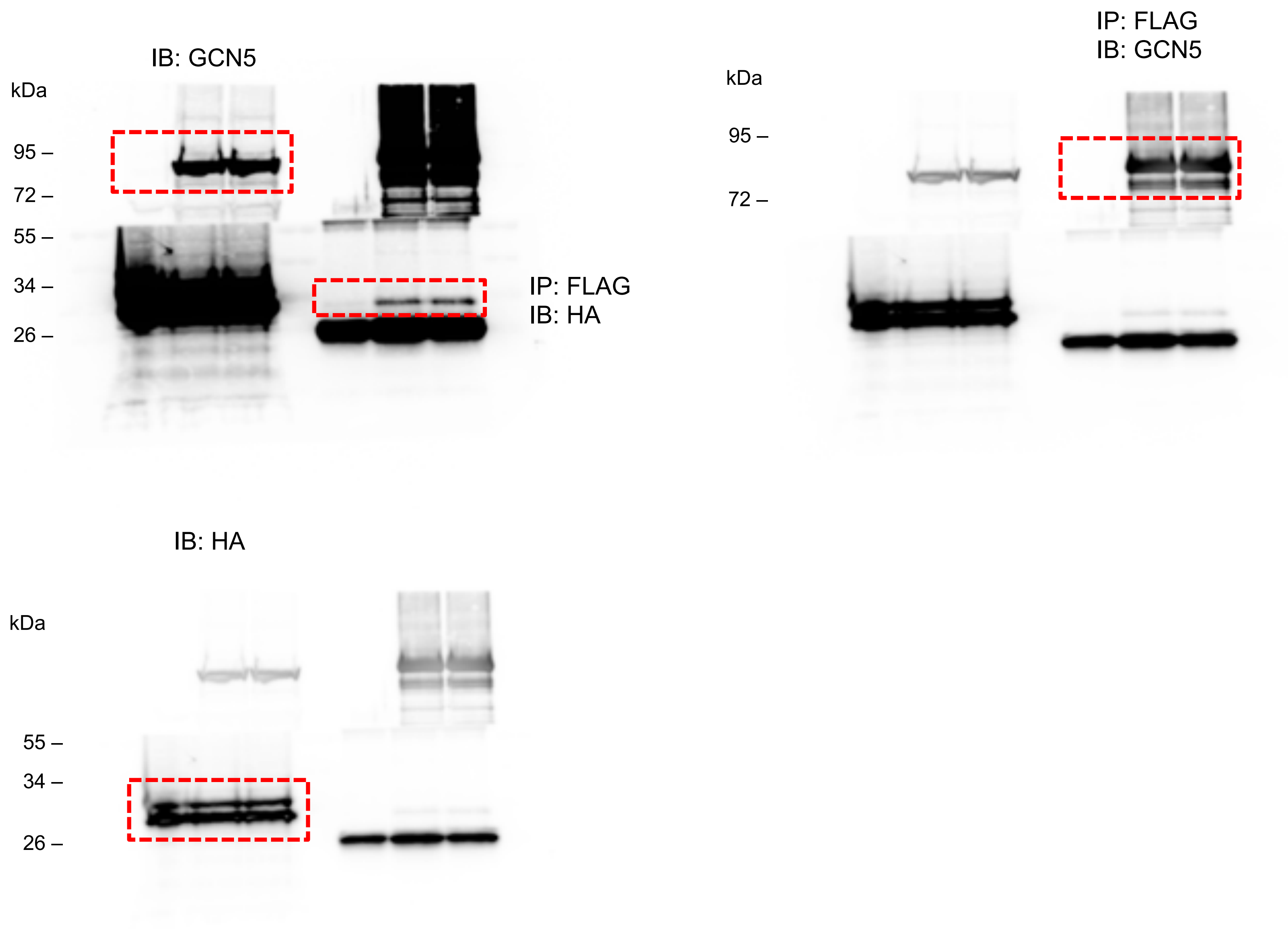

Supplementary Figure S7 (Continued on next page)

Figure 4H

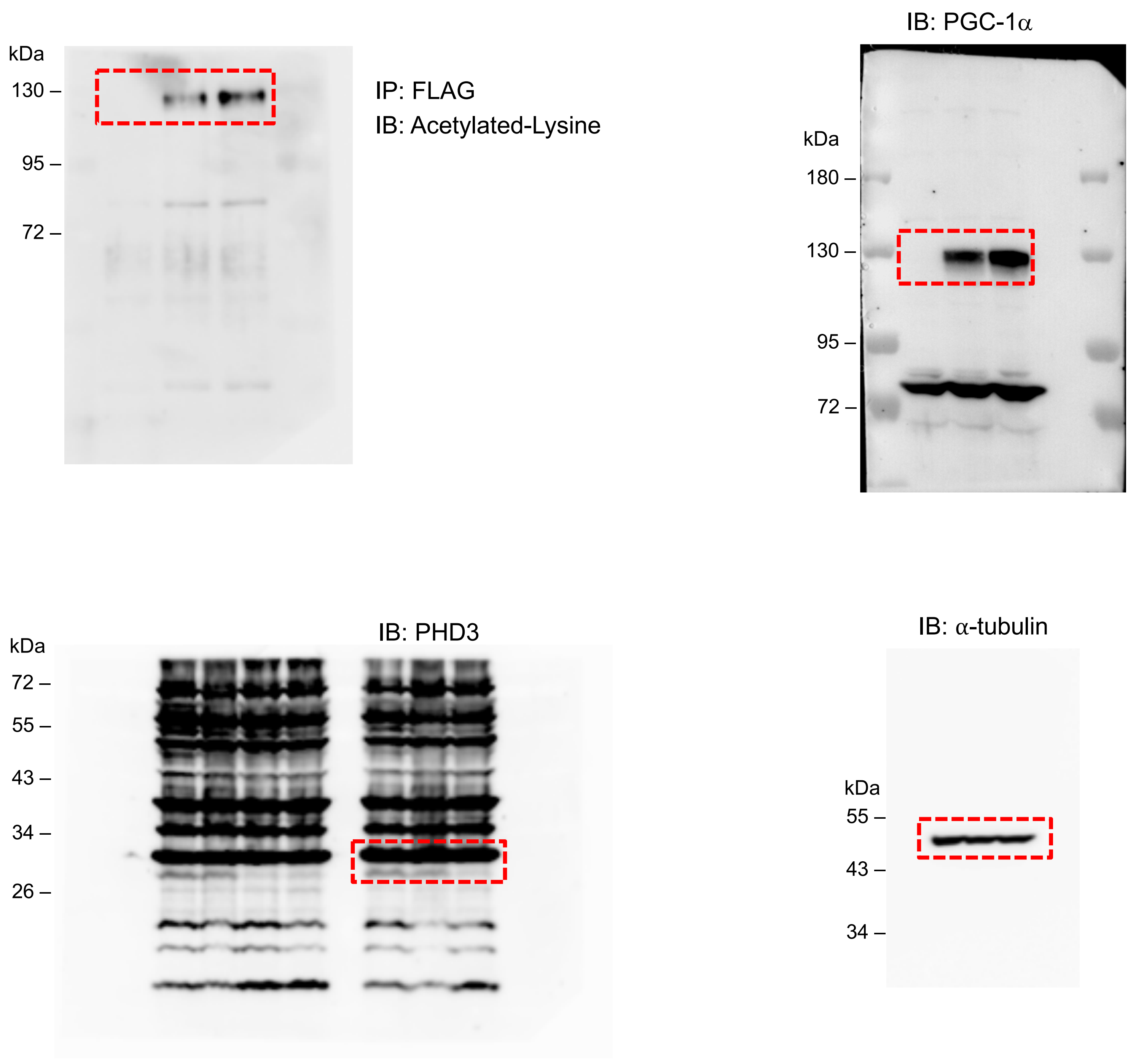

Figure 5A

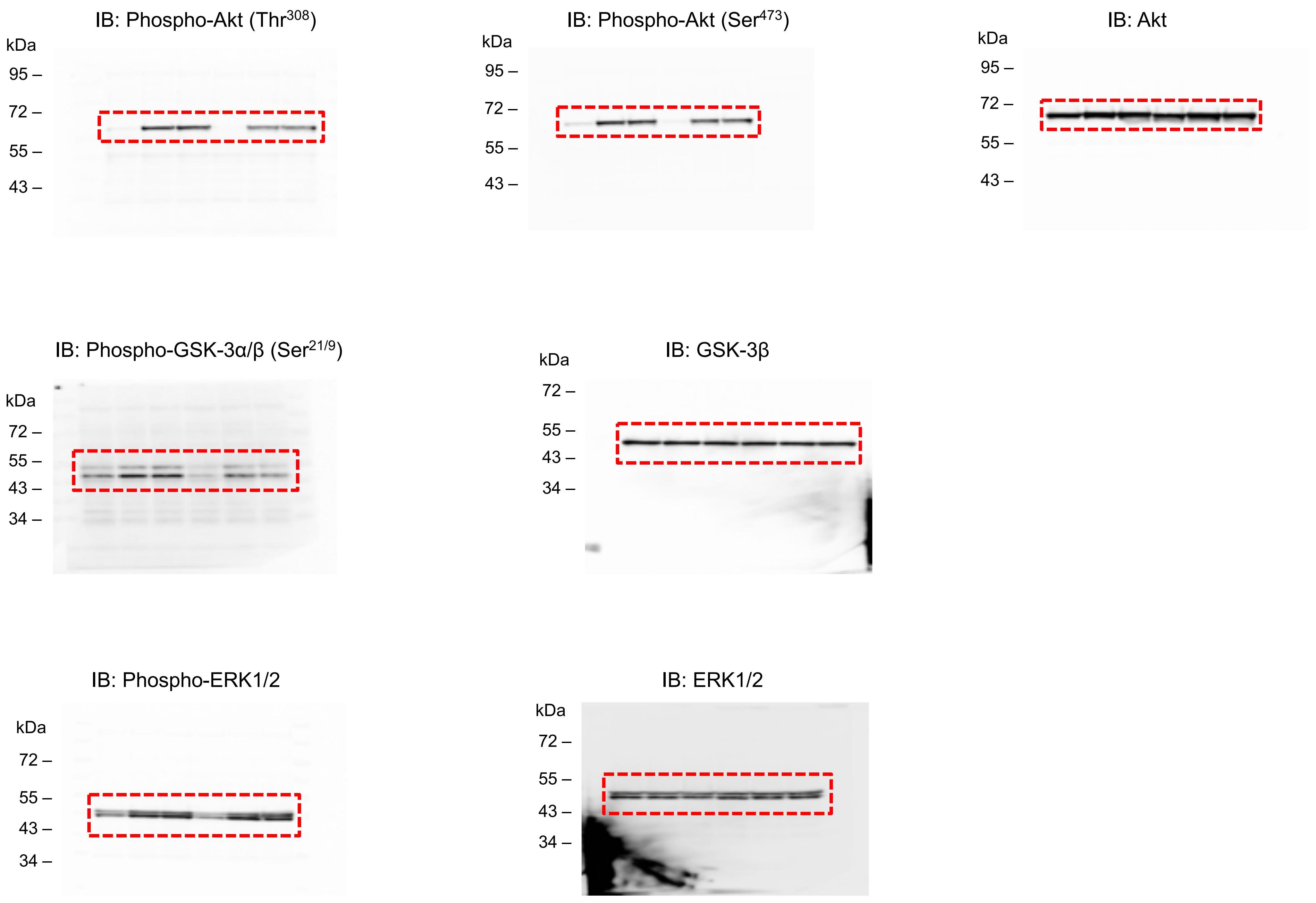

Supplementary Figure S7 (Continued on next page)

Figure 5C

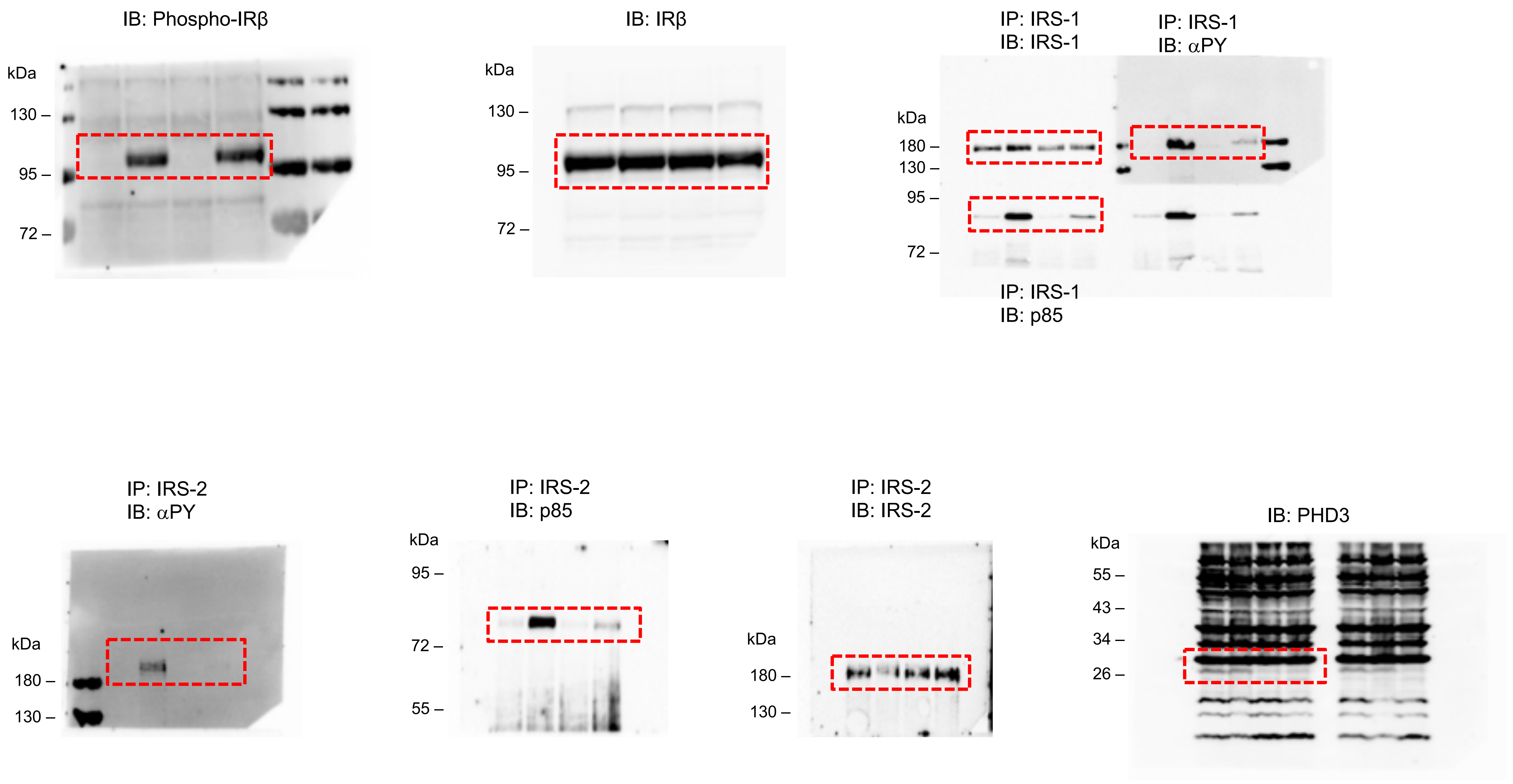

Figure 5E

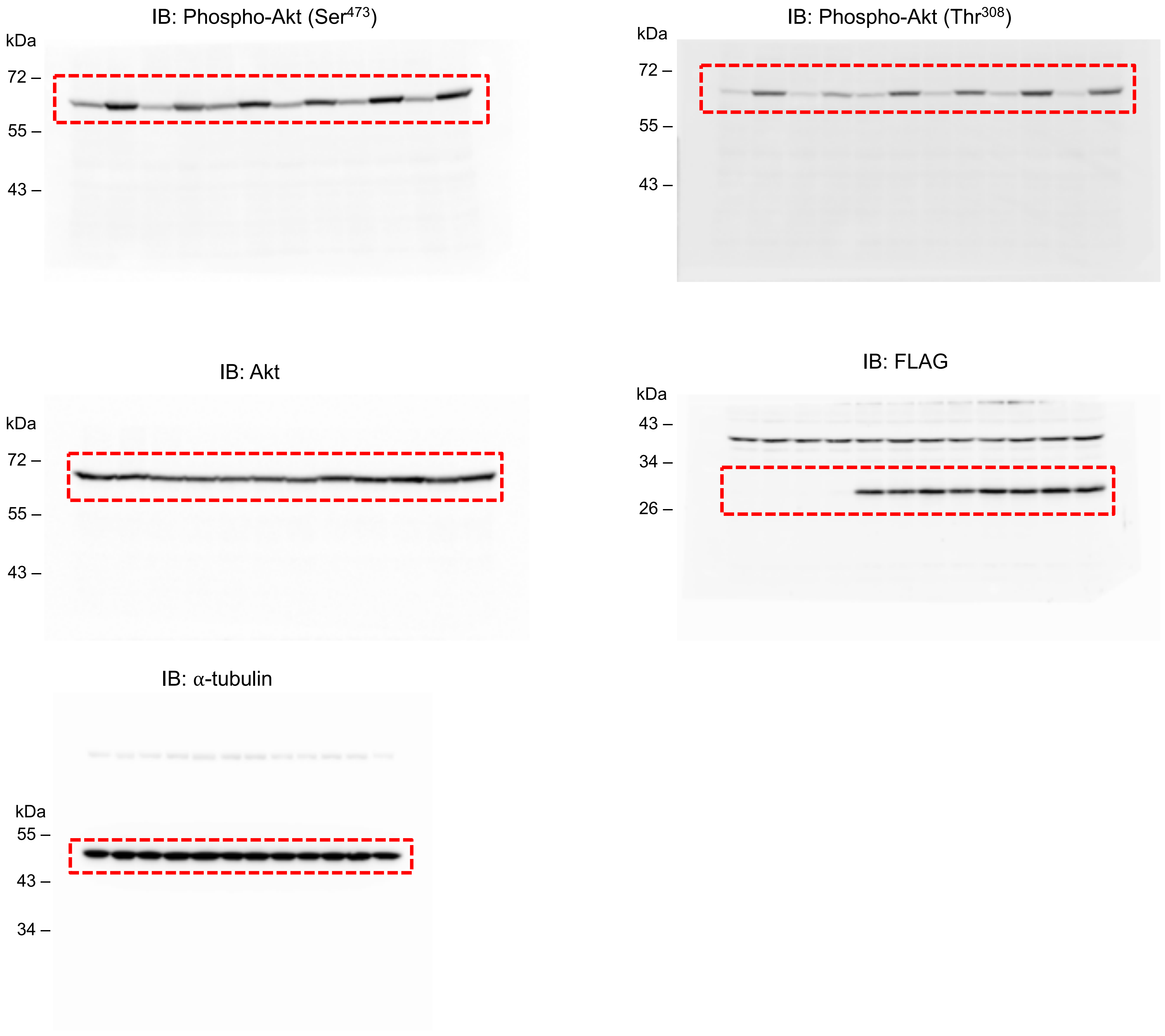

Supplementary Figure S7 (Continued on next page)

Figure 5F

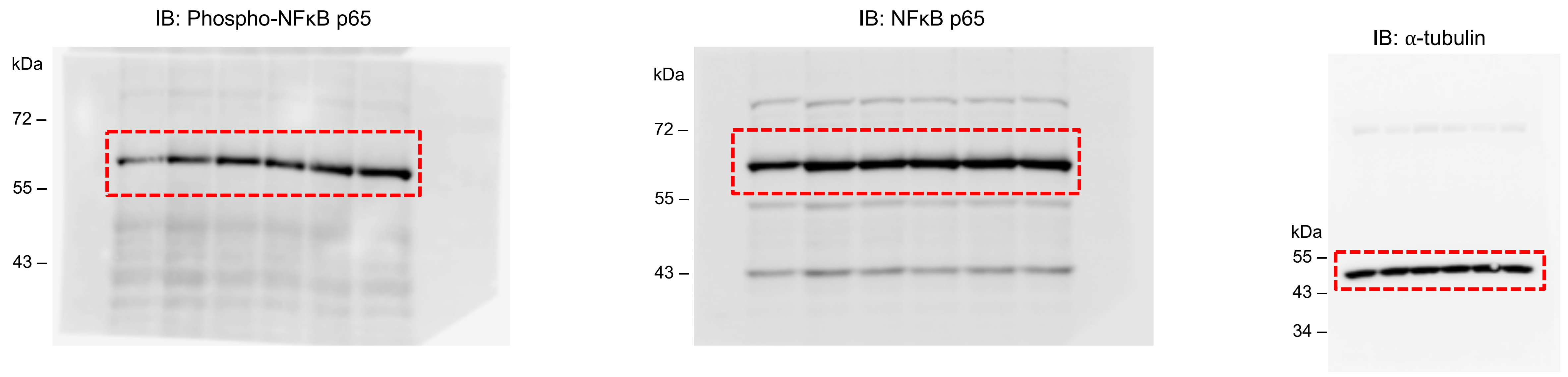

Figure 5G

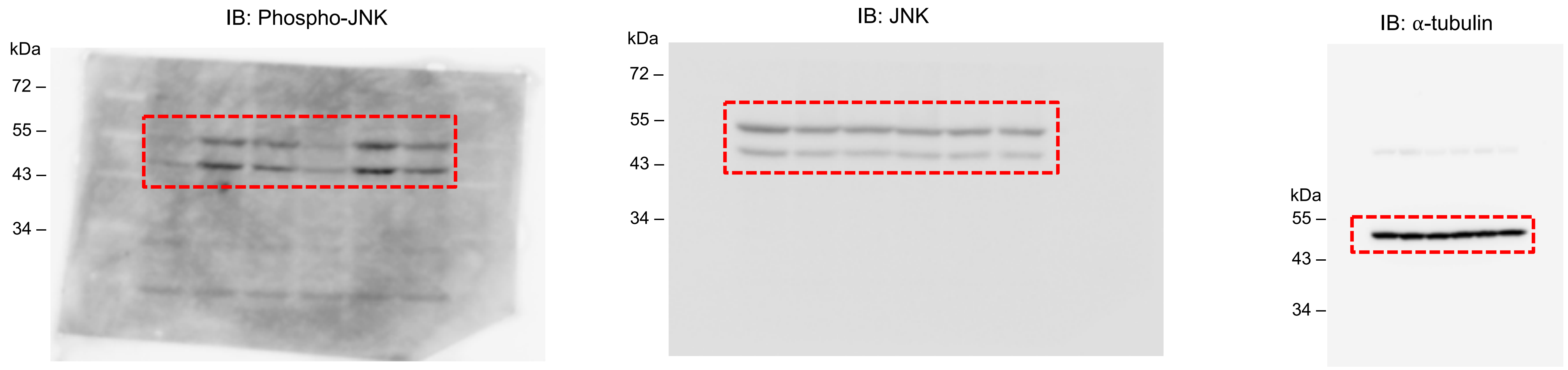

Figure 7A

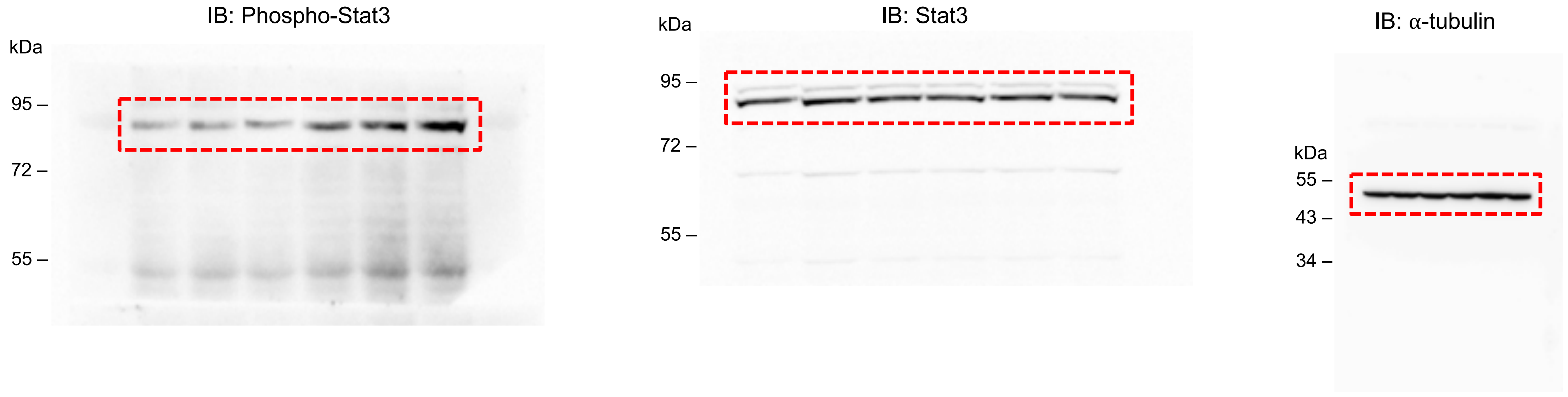

Figure 7B

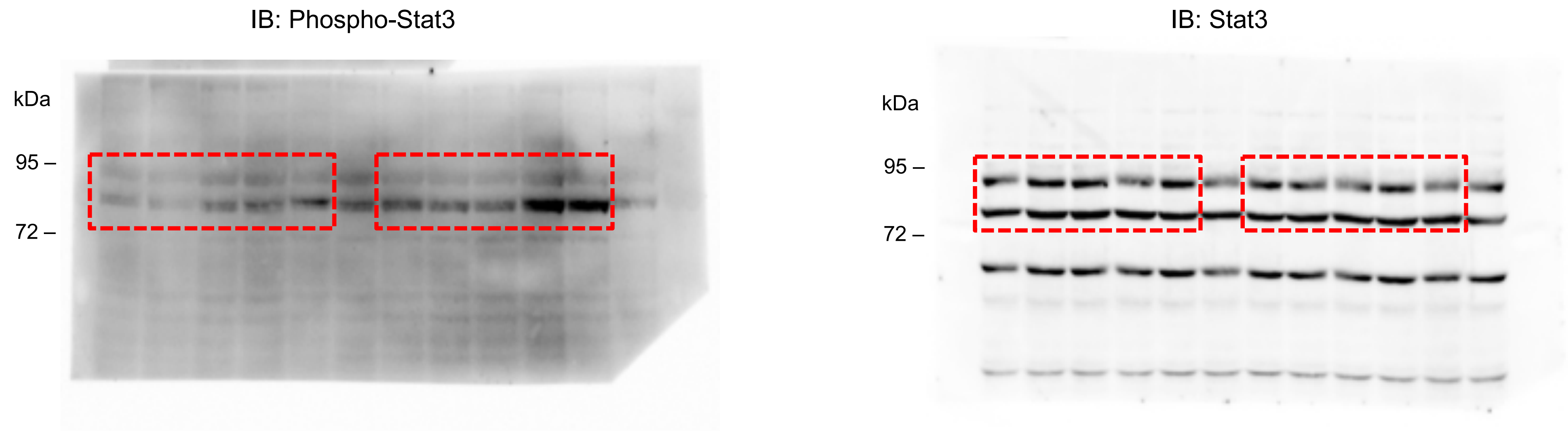

Supplementary Figure S7 (Continued on next page)

Figure 7E

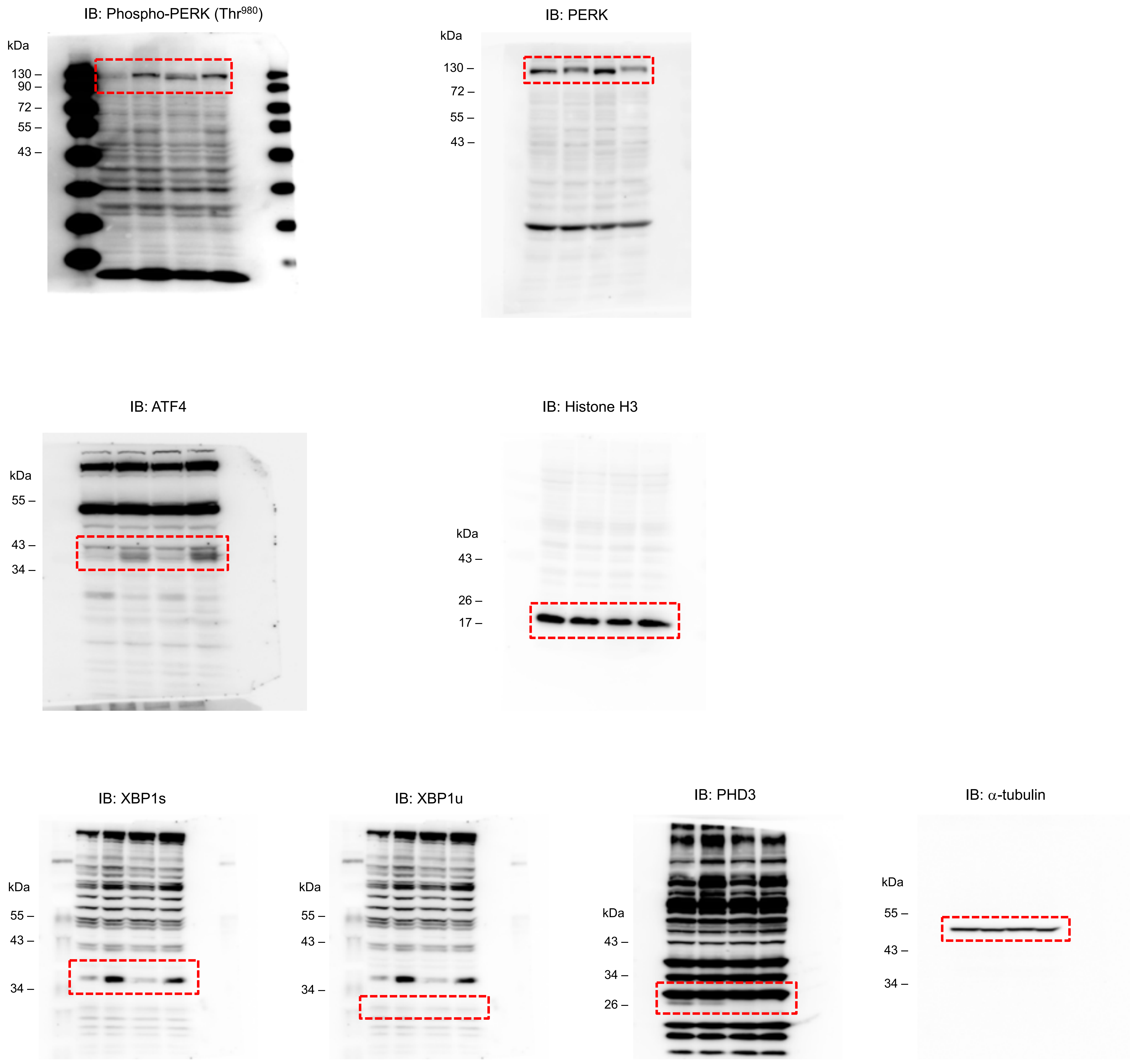

Supplementary Figure S3D

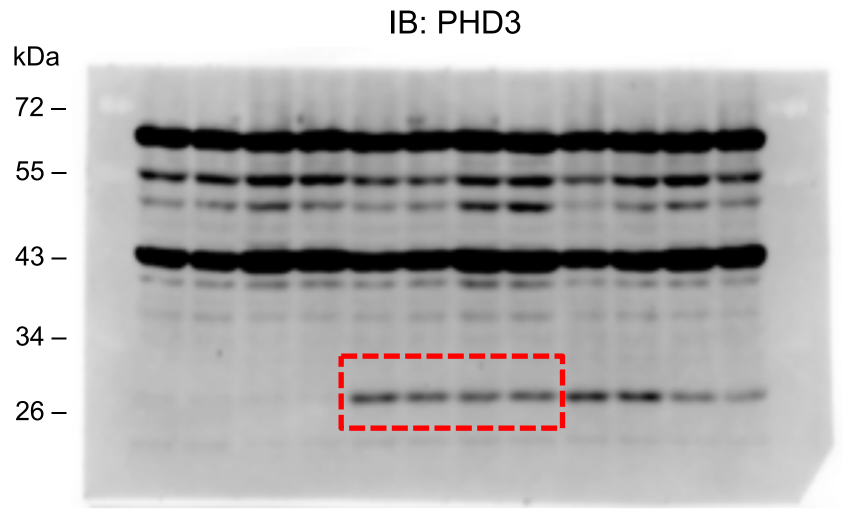

Supplementary Figure S3E

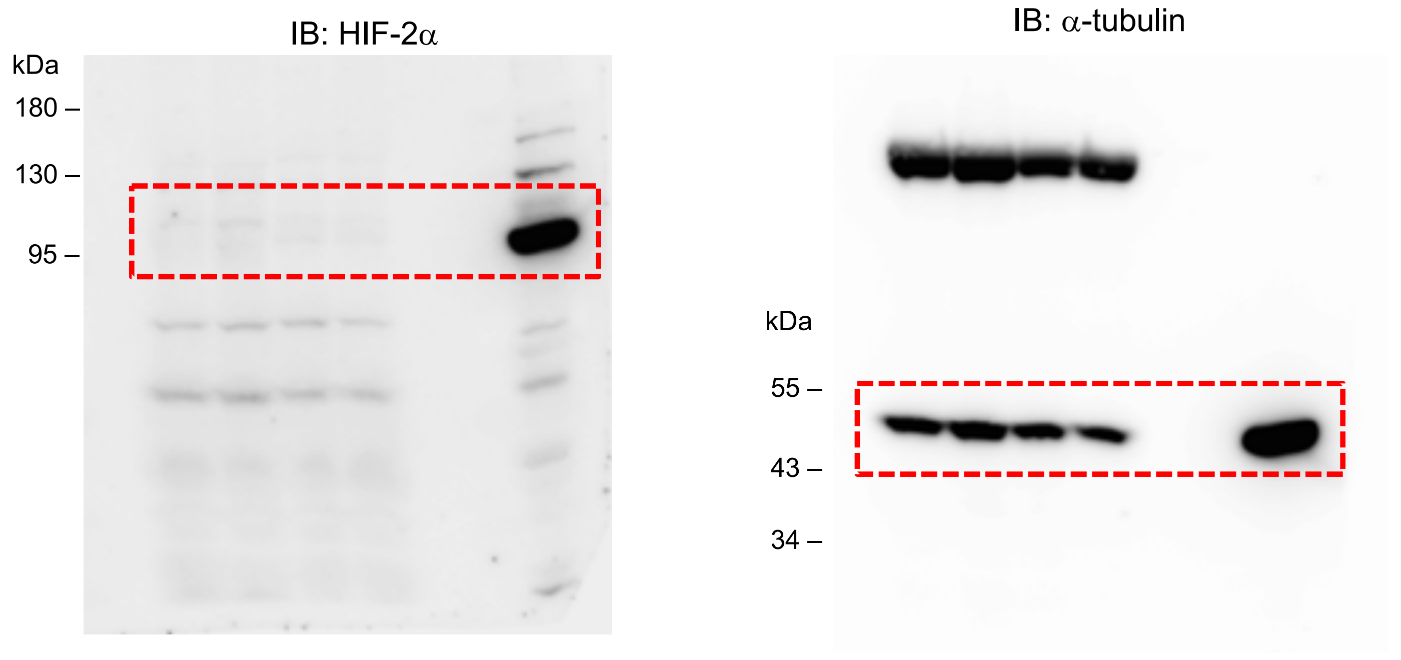

Supplementary Figure S7 (Continued on next page)

Supplementary Figure S5A

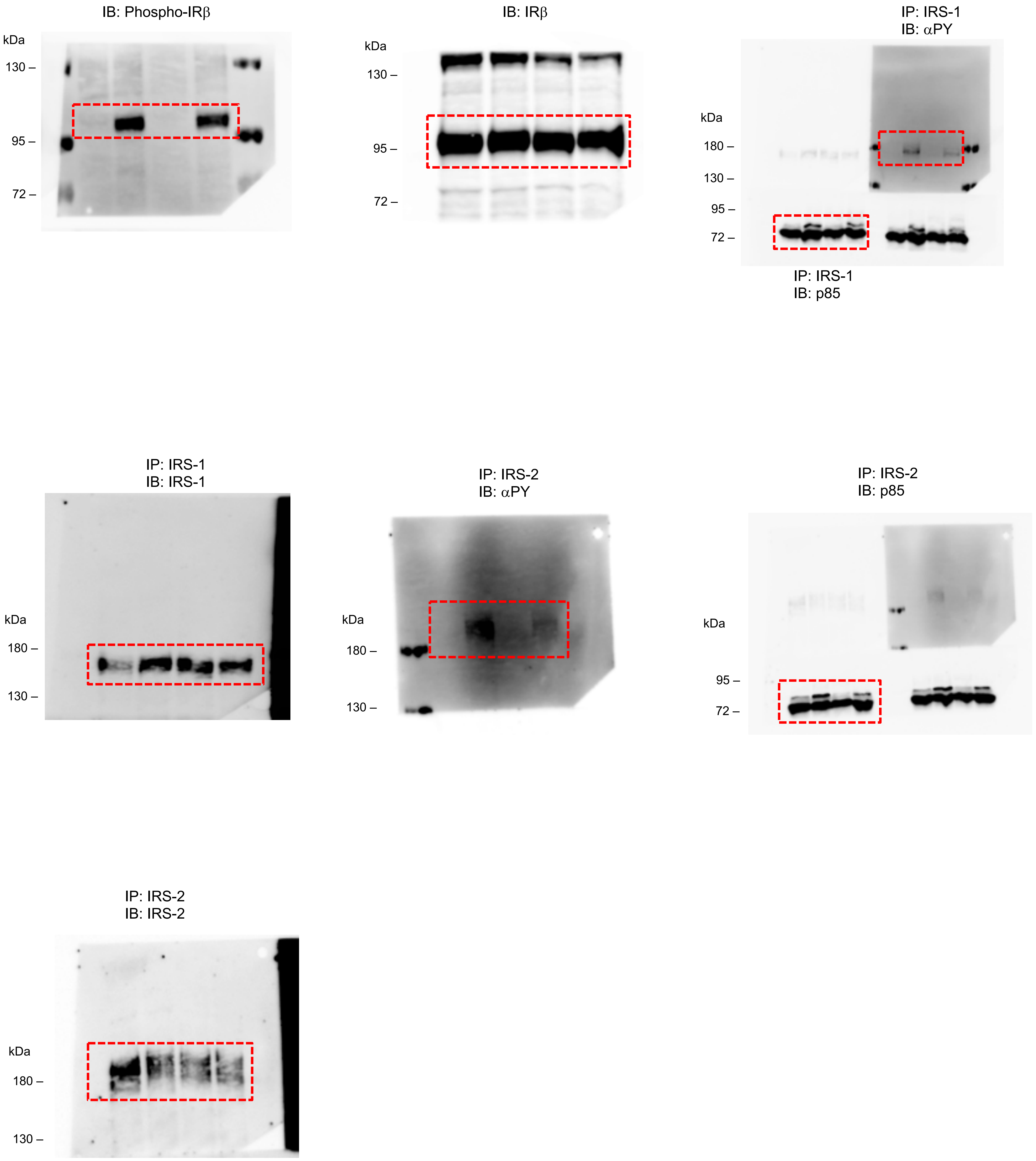

Supplementary Figure S7 (Continued on next page)

Supplementary Figure S5B

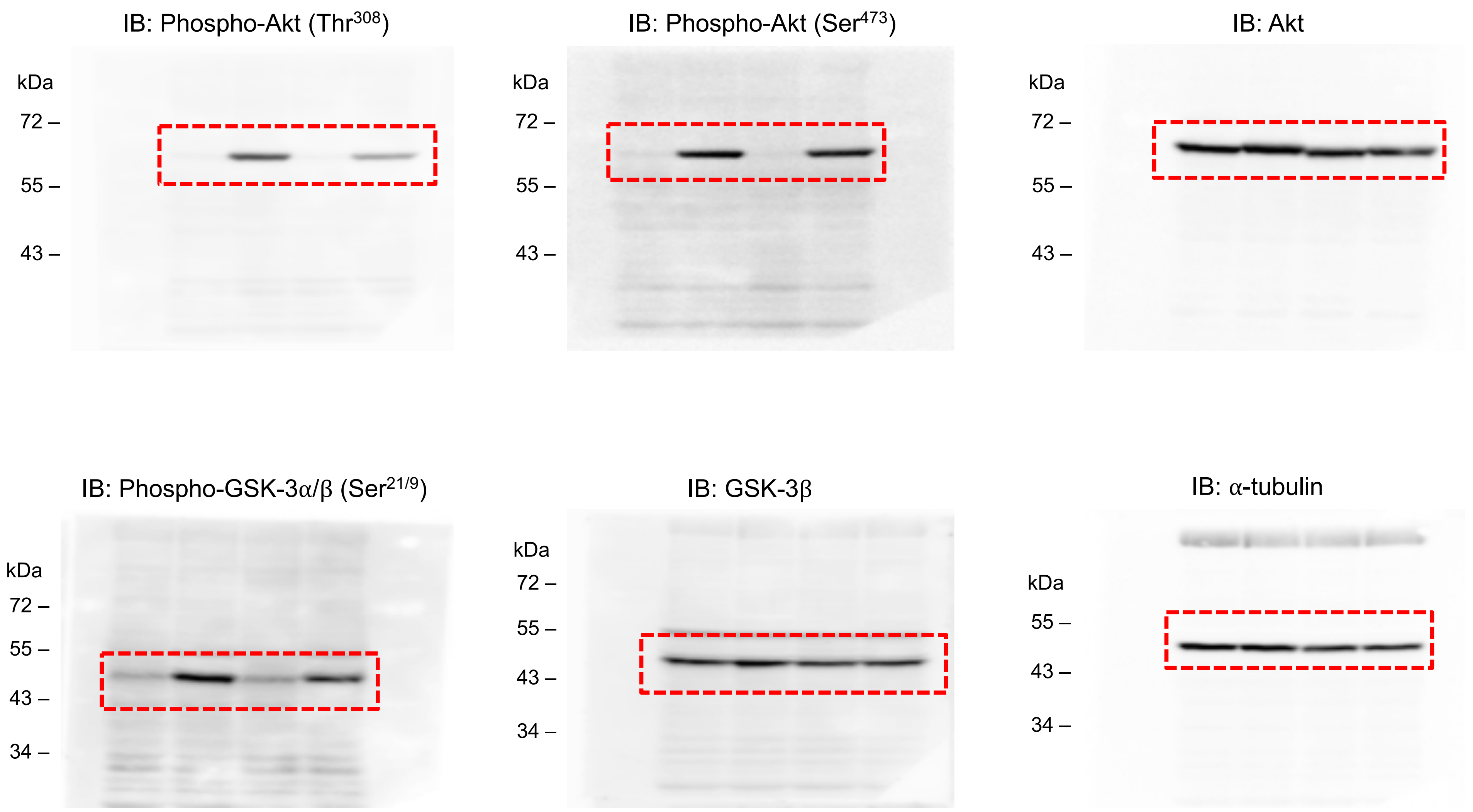

Supplementary Figure S5C

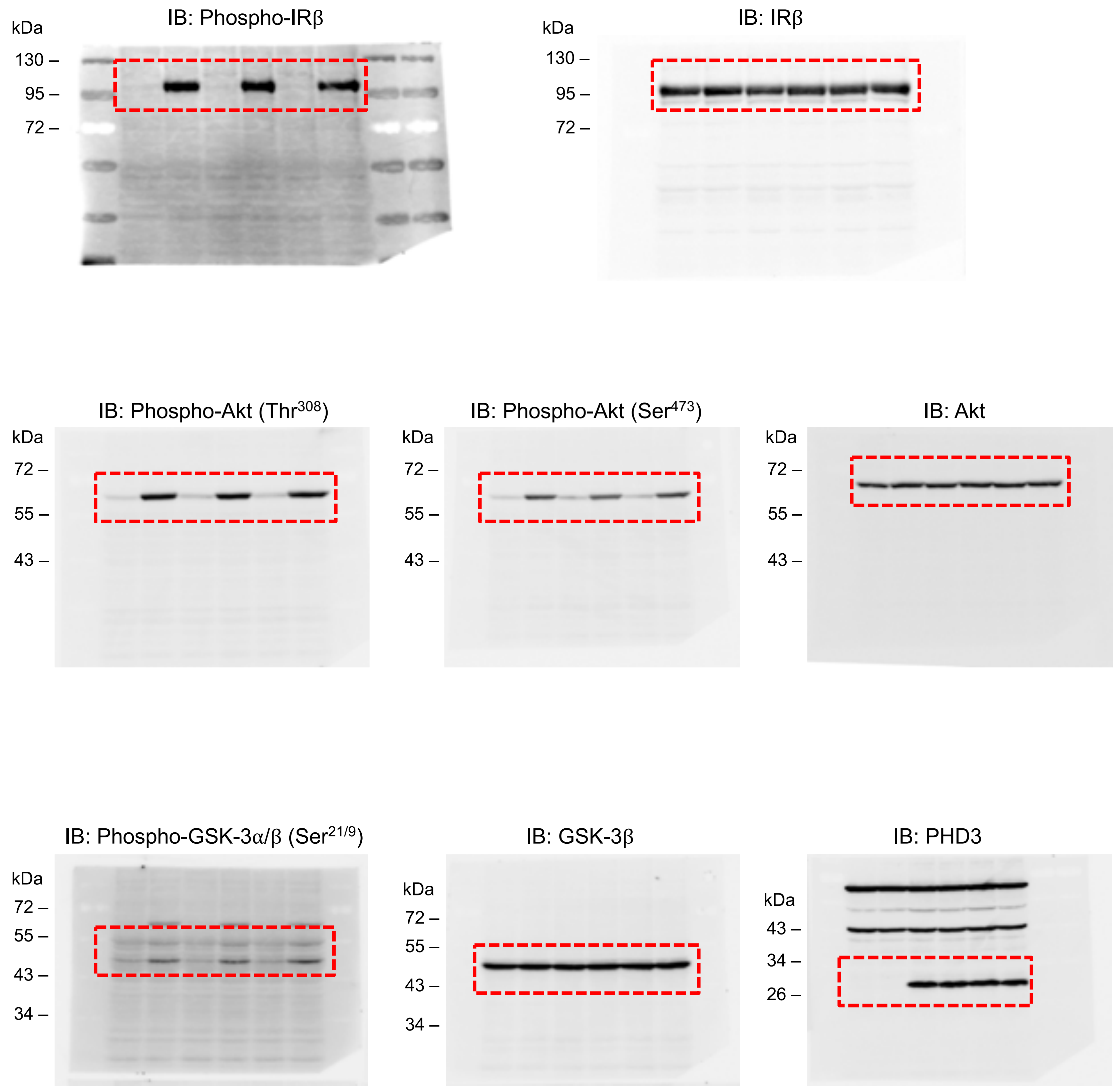

Supplementary Figure S7 (Continued)

Supplementary Figure S5E

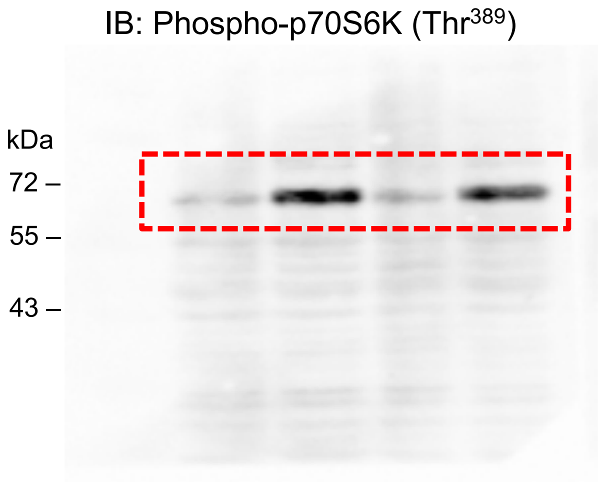

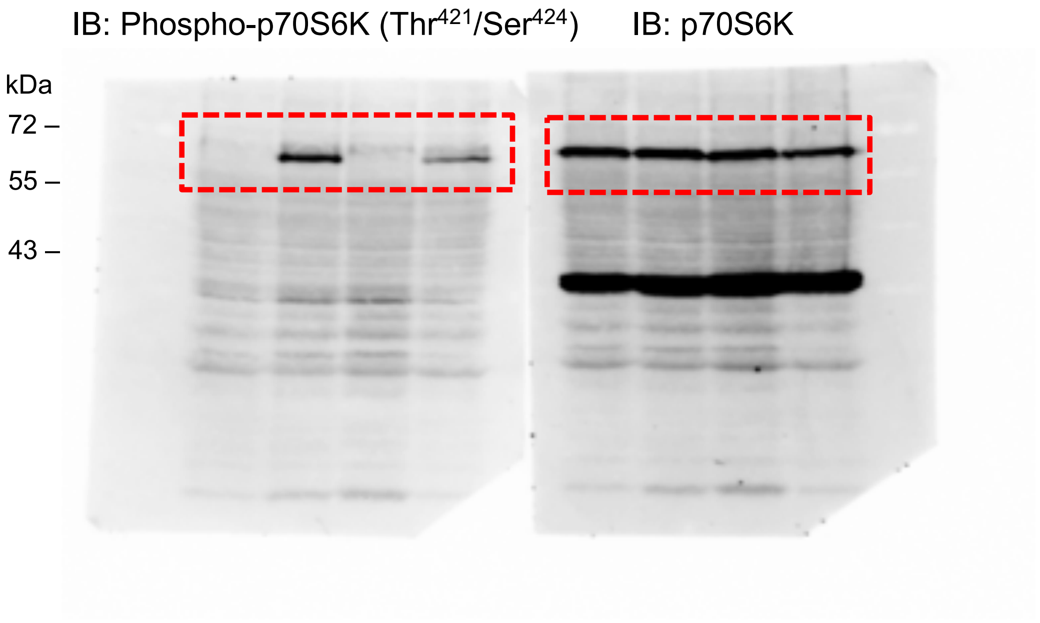

Supplement: Supplementary file 1 — Supplementary information [file 41598_2018_32575_MOESM1_ESM.pdf]
